# Deep Subseafloor Biogeochemical Processes and Microbial Populations Potentially Associated with the 2011 Tohoku-oki Earthquake at the Japan Trench Accretionary Wedge (IODP Expedition 343)

**DOI:** 10.1264/jsme2.ME22108

**Published:** 2023-06-16

**Authors:** Shinsuke Kawagucci, Sanae Sakai, Eiji Tasumi, Miho Hirai, Yoshihiro Takaki, Takuro Nunoura, Masafumi Saitoh, Yuichiro Ueno, Naohiro Yoshida, Takazo Shibuya, James Clifford Sample, Tomoyo Okumura, Ken Takai

**Affiliations:** 1 Super-cutting-edge Grand and Advanced Research (SUGAR) Program, Institute for Extra-cutting-edge Science and Technology Avant-garde Research (X-STAR), Japan Agency for Marine-Earth Science and Technology (JAMSTEC), Yokosuka 237–0061, Japan; 2 Marine Biodiversity and Environmental Assessment Research Center (BioEnv), Research Institute for Global Change (RIGC), Japan Agency for Marine-Earth Science and Technology (JAMSTEC), Yokosuka, Kanagawa Prefecture 237–0061, JAPAN; 3 Research Center for Bioscience and Nanoscience (CeBN), Japan Agency for Marine-Earth Science and Technology (JAMSTEC), Yokosuka 237–0061, Japan; 4 The University Museum, The University of Tokyo, Tokyo 113–0033, Japan; 5 Department of Earth and Planetary Sciences, Tokyo Institute of Technology, Tokyo 152–8551, Japan; 6 Earth-Life Science Institute, Tokyo Institute of Technology, Tokyo 152–8551, Japan; 7 National Institute of Information and Communications Technology, Tokyo 184–8795, Japan; 8 School of Earth and Sustainability at Northern Arizona University; 9 Center for Advanced Marine Core Research, Kochi University

**Keywords:** Japan Trench Fast Drilling Project (JFAST), Integrated Ocean Drilling Program (IODP) Expedition 343, acetogenesis, methanogenesis, coseismic fluid

## Abstract

Post-mega-earthquake geochemical and microbiological properties in subseafloor sediments of the Japan Trench accretionary wedge were investigated using core samples from Hole C0019E, which was drilled down to 851‍ ‍m below seafloor (mbsf) at a water depth of 6,890 m. Methane was abundant throughout accretionary prism sediments; however, its concentration decreased close to the plate boundary decollement. Methane isotope systematics indicated a biogenic origin. The content of mole­cular hydrogen (H_2_) was low throughout core samples, but markedly increased at specific depths that were close to potential faults predicted by logging-while-drilling ana­lyses. Based on isotopic systematics, H_2_ appeared to have been abundantly produced via a low-temperature interaction between pore water and the fresh surface of crushed rock induced by earthquakes. Subseafloor microbial cell density remained constant at approximately 10^5^‍ ‍cells‍ ‍mL^–1^. Amplicon sequences revealed that predominant members at the phylum level were common throughout the units tested, which also included members frequently found in anoxic subseafloor sediments. Metabolic potential assays using radioactive isotopes as tracers revealed homoacetogenic activity in H_2_-enriched core samples collected near the fault. Furthermore, homoacetogenic bacteria, including *Acetobacterium carbinolicum*, were isolated from similar samples. Therefore, post-earthquake subseafloor microbial communities in the Japan Trench accretionary prism appear to be episodically dominated by homoacetogenic populations and potentially function due to the earthquake-induced low-temperature generation of H_2_. These post-earthquake microbial communities may eventually return to the steady-state communities dominated by oligotrophic heterotrophs and hydrogenotrophic and methylotrophic methanogens that are dependent on refractory organic matter in the sediment.

The subseafloor biosphere is characterized by a highly abundant biomass and biodiversity (Morono *et al.*, 2011; Kallmeyer *et al.*, 2012; Hoshino *et al.*, 2020). Scientific ocean drillings have provided insights into the phylogenetic diversity and function of a number of subseafloor environments, such as ultradeep (2.5‍ ‍km below the seafloor) sediments of the Japan Trench forearc basin (Inagaki *et al.*, 2015), oligotrophic pelagic sediments of the South Pacific Gyre (D’Hondt *et al.*, 2015), high-temperature sediments of the Nankai Trough (Heuer *et al.*, 2020; Beulig *et al.*, 2022), deeply-buried ridge flank basalt (Cowen *et al.*, 2003; Lever *et al.*, 2013), highly alkaline serpentinite bodies (Mottl *et al.*, 2003; Kawagucci *et al.*, 2018; Früh-Green *et al.*, 2018), and deep-sea hydrothermal systems and volcanos (Yanagawa *et al.*, 2016; de Ronde *et al.*, 2019; Teske, 2020). These findings revealed that the development of a subseafloor microbial community is closely associated with the energy and nutrient states for growth and survival under the present physical and chemical conditions of these habitats and is affected by genetic and phenotypic adaptation histories to long-term geological and geochemical processes. To clarify a greater spectrum of the global subseafloor microbial ecosystem, further investigations are still required into previously unexplored subseafloor environments, which have different geological and geochemical processes and histories from those examined to date.

The Japan Trench Fast Drilling Project (JFAST) performed Integrated Ocean Drilling Program (IODP) Expedition 343 one year after the 2011 Tohoku-oki earthquake to investigate the subseafloor processes that occurred at the destructive earthquake and during its aftershocks (Chester *et al.*, 2013a, 2013b; Fulton *et al.*, 2013; Ujiie *et al.*, 2013; Rabinowitz *et al.*, 2015; Kodaira *et al.*, 2021). The project drilled the hadal seafloor at a water depth of 6,890 m, reached 851‍ ‍m below seafloor (mbsf), and recovered a core from Hole C0019E (Core C0019E) covering the overlying Japan Trench accretionary prism and plate boundary decollement (Chester *et al.*, 2013a). Drilling and post-drilling operations successfully detected the locations and structures of potential faults associated with a coseismic slip at the hole and a thermal anomaly due to frictional heating (Fulton *et al.*, 2013). Furthermore, the pore-water chemistry of core samples indicated earthquake-induced environmental changes and subsequent microbial responses associated with the mega-earthquake and historical earthquake swarms. Onboard measurements of pore-water mole­cular hydrogen (H_2_) revealed sharp peaks in a vertical profile (Chester *et al.*, 2013a). Coseismic H_2_ generation has been proposed by multiple studies that performed fault zone soil observations (Kita *et al.*, 1980; Wakita *et al.*, 1980), subland fault drilling (Wiersberg and Erzinger, 2008), rock crushing experiments (Kita *et al.*, 1982), and fault sliding experiments (Hirose *et al.*, 2011). The coseismic upwelling of deep subseafloor fluids (*e.g.* Kawagucci *et al.*, 2012; Sano *et al.*, 2014) is another example of earthquake-induced environmental changes with fluid migration. One of the scientific questions for the JFAST expedition was whether an abundant supply of coseismic H_2_ at the mega-earthquake and even historical earthquake swarms affected the subseafloor microbial community compositions and functions inhabiting the accretionary prism and plate boundary decollement, particularly hydrogenotrophic chemolithoautotrophic populations, such as homoacetogens and methanogens (*e.g.* Wu and Lai, 2011).

The present study was primarily conducted to answer this question. We herein describe pore-water and sediment chemistries as well as the composition and functions of the microbial community in Core C0019E. In addition to onboard chemical ana­lyses performed according to the standard IODP protocol and H_2_ measurements (Chester *et al.*, 2013a), detailed onshore investigations, including stable isotope ana­lyses and culture-dependent and -independent microbiological characterizations, were conducted. To address the possible origins of the anomalously abundant H_2_ enrichment at specific depths in the accretionary prism, we also performed an experimental simulation for isotope fractionation associated with H_2_ generation through a low-temperature fluid-rock interaction. These comprehensive ana­lyses imply episodic and steady-state subseafloor geochemical and microbiological processes in the seismically active accretionary wedge of the Japan Trench.

## Materials and Methods

### Sampling

IODP Expedition 343 of D/V *Chikyu* was conducted between April and May 2012 (Fig. S1). Sampling procedures and preliminary results obtained during the expedition, including geological characteristics, physical properties, and chemical ana­lyses of the core, have already been reported (Chester *et al.*, 2013a). A summary of this study is described below.

Twenty-one core sections (C0019E-1R to -21R) were recovered during the expedition. The core was classified into seven lithological units based on color, composition, grain size, and minor lithology. Unit 1 was shallower (176.5–185.2 mbsf; 1R) than the other units (648.0–836.8 mbsf; 2R-21R). Unit 3 (688.5–820.1 mbsf; 4R-16R), which was the longest, had a more terrigenous nature than Units 1 and 2 and contained abundant amounts of pyrite-like grains. Unit 4 (821.5–822.5 mbsf; 17R) consisted of sheared clay, recognized as decollement. Unit 5 (824.0–832.9 mbsf; 18R-20R) represented the underthrust incoming plate, while Units 6 and 7 (832.9–836.8 mbsf; 20R-21R) represented incoming plate sediments. In Unit 3, logging-while-drilling ana­lyses identified a series of low resistivities between 688–701 and 720 mbsf, suggesting the presence of faults. Structural geology also identified numerous fractures, including some potential faults, and the most probable major fault zones were identified at 720 and 820 mbsf (Chester *et al.*, 2013a).

Sampling and analytical procedures on the major geochemical parameters of cations (K^+^, Ca^2+^, Mg^2+^, Mn^2+^, and NH_4_^+^) and anions (Cl^–^, SO_4_^2–^) in pore water and the composition of gas (CH_4_ and H_2_) as well as the solid-phase properties of Total Organic Carbon (TOC), Total Nitrogen (TN), and Total Sulfur (TS) were described in the expedition proceedings (Chester *et al.*, 2013a). The present study used the values reported.

Pore-water was extracted from whole-round cores through a 0.2-μm disposable polytetrafluoroethylene filter by the squeezer assembly. Since most of the recovered core samples were fragmented during the coring operation and exposed to seawater and/or seawater-based drilling fluid during recovery, the pore-water sampling process, such as peeling of the outer layer, may have been insufficient to completely remove seawater contamination from the pristine inner parts of core samples. Sediment squeeze cakes were sealed in plastic bags for shore-based ana­lyses. Regarding gas ana­lyses, approximately 1‍ ‍mL of sediment or deposit was collected with a cut-off plastic syringe or corkscrew and extruded into a 20-mL glass vial containing 3‍ ‍mL of Milli-Q water and a small amount of HgCl_2_ to prevent microbial activity. Onboard H_2_ and hydrocarbon ana­lyses were conducted after heating the vials at 70°C for 30‍ ‍min followed by measurements with gas chromatographs (GL Science GC4000 and Agilent 6890N) equipped with a helium ionization detector (HID) and flame ionization detector (FID), respectively. Vials were then stored in a freezer until onshore isotope ana­lyses. The number of (sub)samples for pore-water (*n*=12) was lower than that for gas species (*n*=52) due to insufficient core recovery.

Subsamples for microbiological ana­lyses were taken from the inner part of a whole round core with a sterilized spatula. Microscopic observations of cell density, a cultivation test, and radioactive isotope tracer incubation experiments were conducted on 12 core samples (1, 4–8, 12–15, and 19–20R) and 16S rRNA gene amplicon sequencing on 9 (3, 6, 9–10, and 12–16). In microscopic observations, approximately 1‍ ‍g of fragments from each sample was placed into a plastic tube and fixed with 3‍ ‍mL of filtered phosphate-buffered saline (PBS; pH 7.2; filtered through a pore size of 0.22‍ ‍μm) containing 4% (w/v) paraformaldehyde at 4°C for 3 h. Fixed samples were stored at –80°C. To extract DNA, ~30‍ ‍g of the fragments from each core was placed into a plastic tube and stored at –80°C until onshore processing. In cultivation ana­lyses, approximately 75‍ ‍mL of core fragments was placed into a 100-mL autoclaved glass bottle and sealed with butyl rubber stoppers in an anaerobic glove chamber. To maintain samples under strict anaerobic conditions, the headspace of the bottles was pressurized with 200 kPa-N_2_, 0.5‍ ‍mL of 5% (w/v) neutralized Na_2_S solution was added, and bottles were stored at 4°C until onshore laboratory treatments.

### Geochemical and microbiological analyses

#### Geochemical analyses

The carbon and hydrogen isotopic compositions of CH_4_, H_2_, and CO_2_ in the headspace of vial subsamples for gas measurements were assessed using isotope ratio mass spectrometers (MAT253 and DELTA^XP^ADVANTAGE; ThermoFisher) at JAMSTEC as previously described (Kawagucci *et al.*, 2010; Okumura *et al.*, 2016). δD_H2_ was quantified when subsamples contained a sufficient amount of H_2_ (*n*=7 out of 52). The concentrations of gas species presented are the values obtained from onboard ana­lyses, whereas the mixing ratios of H_2_ in the headspace gas measured in MAT253 ana­lyses were used to evaluate the magnitude of air-derived H_2_ contamination by a Keeling plot (Keeling, 1958). The hydrogen and oxygen isotope ratios of interstitial H_2_O were measured by cavity-ring-down spectroscopy (L2130-i; Picarro) at Northern Arizona University. Isotope ratios are presented by conventional δ‍ ‍notation in the permil scale. Analytical uncertainties (1-sigma) were within 0.3‰ (δ^13^C_CH4_), 5‰ (δD_CH4_), 10‰ (δD_H2_), 0.5‰ (δ^13^C_CO2_), 0.3‰ (δD_H2O_), and 0.2‰ (δ^18^O_H2O_). Isotope fractionation between two molecules, A and B, is expressed by α as follows:

$${\alpha^{X}}_{A-B}=\left(1000+{\delta^{X}}_{A}\right)/\left(1000+{\delta^{X}}_{B}\right)\text{,}$$

where X represents ^13^C or D.

The sulfur isotopic compositions of sulfate and sulfide in representative samples were assessed by a combination of sulfur conversion to SF_6_ and a ThermoFisher MAT253 mass spectrometer with a dual inlet system at the Tokyo Institute of Technology. Sulfate dissolved in pore-water was initially precipitated as BaSO_4_ by the addition of a 10% BaCl_2_ solution followed by rinsing with acetone to remove native sulfur. Dried BaSO_4_ was converted to Ag_2_S using the Kiba reduction method (Sasaki *et al.*, 1979). Sulfide-bearing squeezed cakes of sediments were dried and powdered using an agate mill. Powdered samples (2.0–5.5 g) were ultrasonically washed and soaked in 10% NaCl solution for 24 h. Samples were then rinsed with distilled water and centrifuged to remove soluble sulfate. The residue was washed and soaked with acetone for 24‍ ‍h to dissolve native sulfur and then rinsed with distilled water and centrifuged. The residue was dried at room temperature for >24‍ ‍h and sulfide was extracted using a modified method from Hsieh and Shieh (1997). The dried residue and alkaline Zn trap were placed in a 500-mL glass bottle. The bottle was purged with N_2_ and the sample was reacted with 20‍ ‍mL of 5 M HCl followed by 20‍ ‍mL of chromium (II) solution at room temperature for >48‍ ‍h (Ueno *et al.*, 2008). Chromium (II)-reducible sulfur (CRS) was reduced to H_2_S and precipitated as ZnS in a 20-mL alkaline Zn trap. In this extraction procedure, acid volatile sulfur was also reduced to H_2_S and precipitated as ZnS; however, our preliminary ana­lyses showed that negligible acid volatile sulfur was present in the samples (data not shown). ZnS was converted to Ag_2_S by a reaction with 0.1 M AgNO_3_ solution, cleaned via repeated centrifugation with distilled water, and dried at 70°C for >12 h. Ag_2_S was reacted with excess F_2_ at 300°C in a nickel reaction tube overnight to produce SF_6_, which was purified using a cryogenic technique and gas chromatography. The multiple sulfur isotopic composition is presented using delta notation:

δxS=Sx/S32sample/Sx/S32reference-1×1000(x=33 or 34),

where (^x^S/^32^S)_sample_ and (^x^S/^32^S)_reference_ are the isotope ratios of the sample and reference material, respectively.

ΔS33=δ33S-1+δ34S/10000.515-1×1000.

The analytical reproducibilities of the δ^34^S and Δ^33^S values, as assessed by replicate ana­lyses of the international reference material IAEA-S1, were ±0.3‰ and ±0.01‰(1σ), respectively. The sulfur isotope effect during sulfate reduction is described as follows:

ε34=1000∙1-S34/S32sulfide/S34/S32sulfate

λ33=lnS33/S32sulfide/S33/S32sulfate/lnS34/S32sulfide/S34/S32sulfate,

where (^x^S/^32^S)_sulfide_ and (^x^S/^32^S)_sulfate_ (x=33, 34) are the sulfur isotope ratios of sulfide and sulfate, respectively.

In textural observations of sulfide minerals near the fault zones in Unit 3, subsamples were collected from 7R02 and 13R02, which had localized high intensity parts that were observed by onboard X-ray and CT scans (Chester *et al.*, 2013a). Collected samples were embedded in LR White resin after dehydration with an ethanol series; 70, 80, 90, and 99%. Embedded samples were polished, coated with carbon using a vacuum evaporation system (JEE-420; JOEL), and observed by a field emission Scanning Electron Microscope (SEM) (JSM-6500F; JEOL) at 15kV in Kochi University, with Energy Dispersive X-ray spectrometry (EDS) for the elemental distribution mapping.

#### Microbiological ana­lyses

To investigate cell density in core samples, fixed samples were washed with 1× PBS, resuspended in 1× PBS-ethanol (1:1) solution, and stored at –20°C. Just before the experiment, each sample (100‍ ‍μL) was diluted with 1× PBS (900‍ ‍μL), and the diluted sample was sonicated for 20‍ ‍s using a UH-50 ultrasonic homogenizer (SMT). Approximately 5‍ ‍mL of filtered (0.22‍ ‍μm) 1× PBS was placed into the filter tower prior to the addition of the diluted sample to ensure an even distribution of cells on the filter. The membrane was stained with SYGR green I (1/40‍ ‍[v/v] SYBR green I in TE) at room temperature for 10‍ ‍min in the dark. The membrane was rinsed with 5‍ ‍mL of TE buffer to remove excess dye. Filters were examined under epifluorescence using the phase-contrast microscope BX51 (Olympus). The average total cell count was obtained from more than 100 microscopic fields.

DNA extraction from subsamples was performed using the PowerMAX Soil DNA isolation kit (MoBio Laboratories). Samples were incubated at 65°C for 5‍ ‍min before mechanical shaking for 10‍ ‍min with a ShakeMaster (BioMedical Science), whereas subsequent steps were performed according to the manufacturer’s protocol. Extracted DNA was stored at –80°C. 16S rRNA gene fragments were amplified using the universal primer set Uni530F-U907R (Nunoura *et al.*, 2012), and amplified 16S rRNA gene fragments were purified by agarose gel electrophoresis and the Min Elute PCR purification kit (GIAGEN). Sequence libraries were constructed using the Ion Xpress^TM^ Plus Fragment Library Kit (Life Technologies), and amplicon sequencing was performed on an Ion Torrent PGM sequencer (Life Technologies) equipped with an Ion 314 chip using 400-base read length chemistry by the Ion PGM™ Template OT2 400 Kit and Ion PGM^TM^ Sequencing 400 Kit (Life Technologies). Single-end reads from amplicon libraries were trimmed and filtered using PRINSEQ v0.20.4 with the following parameters: -trim_qual_left 20, -trim_qual_right 20, and -min_len 100. (Schmieder and Edwards, 2011). PCR primers were removed from the processed sequence using Cutadapt v1.10 (Martin, 2011). Cleaned sequences were analyzed using the QIIME2 v2019.4.0 pipeline (Bolyen *et al.*, 2019). Between 77,105 and 146,575 high-quality SSU rRNA gene sequences were obtained, and 77,000 randomly subsampled sequences were used in further ana­lyses. Operational taxonomic units (OTUs) were constructed using a 97% similarity threshold. The taxonomic assignment of OTUs was conducted using the QIIME2 plugin feature-classifier classify-sklearn against the SILVA 138 database (Quast *et al.*, 2013). OTUs presumed to be laboratory contaminants and members of human biomes were excluded from further ana­lyses as previously reported (Nunoura *et al.*, 2016), which included members affiliated with the order *Lactobacillales*, the families *Corynebacteriaceae*, *Micrococcaceae*, *Propionibacteriace*, *Enterobacteriaceae*, *Pseudomonadaceae*, and *Xanthomonadaceae*, and the genera *Leucobacter*, *Streptomyces*, *Staphylococcus*, *Brevundimonas*, *Bradyrhizobium*, *Methylobacterium*, *Pseudochrobactrum*, *Paracoccus*, *Sphingomonas*, *Aquabacterium*, *Burkholderia*, *Cupriavidus*, *Delftia*, *Diaphorobacter*, *Paucibacter*, *Providencia*, *Acinetobacter*, and *Pseudomonas*. Several degenerate oligonucleotide primer sets, ME1/ME2 (Hales *et al.*, 1996), mcrIRD, ANME-1 mcrI (Lever and Teske, 2015), and ME3MF/ME2r’ (Hales *et al.*, 1996; Nunoura *et al.*, 2008) were used to amplify the genes encoding methyl coenzyme M reductase (*mcrA*), thereby expanding phylogenetic coverage with the amplification conditions described in each study. To elucidate the taxonomic characteristics of the isolated pure cultures, DNA extraction, PCR amplification, and sequencing procedures were performed as previously described (Sakai *et al.*, 2019). Amplicon sequence data were deposited into the DNA Data Bank of Japan nucleotide sequence database (DDBJ) Sequence Read Archive under DRA013695. All data were registered under BioProject ID PRJDB13255.

The population density of cultivated microorganisms represented by various physiological and metabolic characteristics was estimated by a series of quantitative cultivation tests (serial dilution cultivation method). To survey members capable of homoacetogenesis and methylotrophic/hydrogenotrophic methanogenesis, core fragments were cultivated at 20°C in MMJ medium (Takai *et al.*, 2002) containing CH_3_NH_2_ (5‍ ‍mM) and acetate (5‍ ‍mM) under an atmosphere of H_2_/CO_2_ (80:20‍ ‍[v/v]). Approximately 0.5‍ ‍g of each core sample was resuspended in 5‍ ‍mL of sterile MJ synthetic seawater (Takai *et al.*, 1999) containing 0.05% of Na_2_S, and this was used as an inoculum. Five hundred microliters of slurry was inoculated into 15-mL tubes containing 5‍ ‍mL of MMJ medium, and 500‍ ‍μL of the medium was then serially transferred to another tube for the next dilution step. The positive dilution tube was subjected to dilution-to-extinction to obtain a pure culture. GenBank/EMBL/DDBJ accession numbers for the 16S rRNA gene sequences of *Acetobacterium* sp. strain JF_A and *Methanolobus* sp. strain JF_M are LC721303 and LC721304, respectively.

The potential microbial metabolic activities of hydrogenotrophic/methylotrophic/aceticlastic methanogenesis and homoacetogenesis were examined by the cultivation of core fragments with each of the radioactive isotope tracers (Tasumi *et al.*, 2015; Kawagucci *et al.*, 2018). Approximately 1‍ ‍g of core fragments was placed into a 30-mL sterilized glass vial followed by the addition of ~10‍ ‍mL artificial seawater medium (0.5 M NaCl, 10‍ ‍mM NaHCO_3_, 0.001% resazurin, and 0.05% Na_2_S) for slurry. The headspace of sealed slurry was purged by N_2_ (or CH_4_ for the methanotrophy test), and hydrogen gas was added only for the samples with ^14^C-bicarbonate, resulting in a final concentration of 1%. The ^14^C-labeled reagents of the possible metabolic substrate (NaH^14^CO_3_, ^14^CH_4_, ^14^CH_3_^14^COOH, and ^14^CH_3_NH_2_) were individually added to each vial in order to set the final radioactivity level to 0.5 MBq per bottle. After the incubation of slurry at 20°C for one month, the ^14^C contents of CH_4_, CO_2_, and acetate in the vials were assessed by gas chromatography (Shimadzu GC-2014; Shimadzu) equipped with the radioisotope detector Raga Star (Raytest) and liquid chromatography (Agilent 1260 Infinity LC system; Agilent) coupled to an online radio flow detector (Ramona Star; Raytest).

### Experiments and calculations

#### Low-temperature experiments

To simulate the isotope effect associated with H_2_ generation through a water-rock reaction at a low temperature, we conducted a reaction between granular zerovalent iron and water, generally known as anaerobic corrosion, hereafter called the Fe-H_2_O reaction. Although H_2_ generation in nature involves Fe(II) in minerals, not Fe(0), the amount of H_2_ experimentally generated through the Fe(II)-H_2_O reaction at a low temperature was shown to be limited (Mayhew *et al.*, 2013), which practically prohibited assessments of the isotope fractionation factor. Therefore, the Fe-H_2_O reaction was adopted in the present study. Briefly, approximately 1‍ ‍g of autoclaved reagent-grade granular iron was placed into a 30-mL glass vial containing 5‍ ‍mL of pure water, δD_H2O_ of which was manipulated by the addition of D_2_O. After purging the vial with pure He, the vial was sealed and stored at 4, 25, or 55°C. Generated H_2_ reached more than 100 ppmv of the headspace of the vial within two weeks, and its δD_H2_ value was measured. In this experiment, possible contamination by air, which contains 0.5 ppmv H_2_ with a δD_H2_ value of +150‰ (Gerst and Quay, 2000), was negligible against experimentally generated H_2_.

#### Thermodynamic calculation

The dependence of the activity of dissolved species on the Gibbs free energy of possible metabolic reactions was estimated based on conventional methods using the B-dot activity model (McCollom and Shock, 1997; Amend *et al.*, 2011; Shibuya *et al.*, 2016). Three chemoautotrophic metabolic reactions involving H_2_ and CH_4_ were considered as follows:

4H_2_+CO_2_=CH_4_+2H_2_O

4H_2_+2CO_2_=CH_3_COOH+2H_2_O

CH_4_+CO_2_=CH_3_COOH

The values of Gibbs free energy were computed according to the equation:

$${∆\;G}_{r}={∆\;G}_{r}^{{}^{\circ}}+{RT\;\ln\;Q}_{r}\text{,}$$

where ${∆\;G}_{r}^{{}^{\circ}}$ represents the standard Gibbs energy of the reaction, *ΔG_r_* free energy, *T* the temperature in Kelvin, *R* the universal gas constant, and *Q_r_* the activity product of the species involved in the reaction. The values of ${∆\;G}_{r}^{{}^{\circ}}$ for the redox reactions were calculated with SUPCRT92 (Johnson *et al.*, 1992). All calculations were conducted assuming the conditions of 25°C and 500 bar. Geochemical parameters given as constant values through the calculations are pH (7.9), ΣCO_2_ (50‍ ‍mM), and [CH_4_] (5‍ ‍mM) according to the observed values at Unit 3. The ΣCH_3_COOH value of 1‍ ‍mM is given for homoacetogenesis to simulate the metabolic potential under acetate-rich conditions. ΣCH_3_COOH values of 1 and 0.001‍ ‍mM are given for acetoclastic methanogenesis to simulate the transition of metabolic potentials along with acetate consumption.

## Results

### Pore-water chemistry

**Fig. 1.**
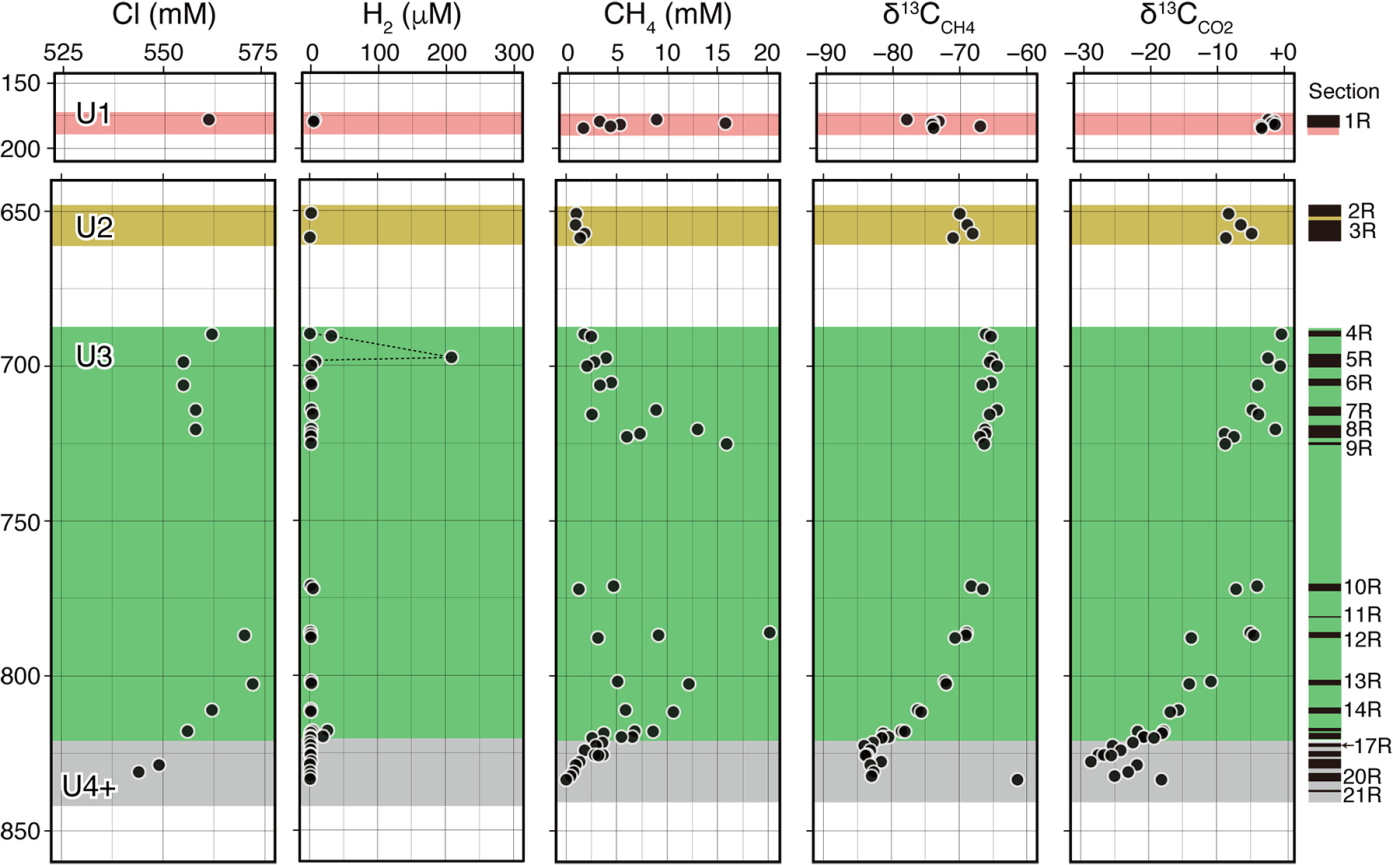
Subseafloor vertical profiles of representative parameters. The vertical profiles of (a, b, and c) the concentrations of Cl^–^, H_2_, and CH_4_ and (d and e) the carbon isotope ratios of CH_4_ and CO_2_ are shown. The corresponding depths of core sections are shown by black horizontal bars with section names on the right side. Background color shades represent lithological units, while U4+ includes Units 4–7.

Geochemical data are presented in Supplementary Table S1. Fig. 1 shows the vertical profiles of the representative major ion Cl^–^, gas concentrations (CH_4_ and H_2_), and carbon isotope ratios of CH_4_ and CO_2_ of C0019E. The average Cl concentration in samples (559±7.5‍ ‍mM) was similar to that in seawater (560‍ ‍mM), which demonstrated that pure-water contamination was negligible during the sampling and pore-water extraction processes. Sulfate concentrations measured from Unit 1–3 and Unit 5 samples ranged between 3–11 and 16–22‍ ‍mM, respectively. The significant amount of sulfate detected in deep subseafloor sediments appeared to be irregular because microbial sulfate reduction coupled with organic matter decomposition completely consumes sulfate in sedimentary environments through geological time. The significant amount of sulfate at these depths may have been due to contamination by seawater sulfate during sample recovery and processing (see Materials and Methods).

Sulfate isotope ratios were used as tracers of the origin and behavior of sulfur-bearing compounds. Only the two core samples (19R-1 and 20R-1) collected in Unit 5 below decollement were the least fragmented and provided the least contaminated pore-water samples (Chester *et al.*, 2013a). The δ^34^S and Δ^33^S values of sulfate in the 1R-1 (Unit 1), 8R-2 (Unit 3), and 20R-1 (Unit 5) samples ranged between +19.7‰ and +23.9‰ and between +0.053‰ and +0.061‰, respectively (Supplementary File S1). These ranges were similar to the δ^34^S and Δ^33^S values of seawater sulfate (*ca.* +21.3‰ and +0.050‰, respectively) (Ono *et al.*, 2012; Tostevin *et al.*, 2014). Therefore, pore-water sulfate appeared to be derived from seawater and sulfur isotope ratios were not modified from the original values. Since a large amount of sulfate was previously reported at the depth of the oceanic basement below decollement (Gieskes *et al.*, 1990, 1993; Hensen and Wallmann, 2005), pore-water sulfate in 20R-1 may have been derived from the subseafloor environment.

**Fig. 2.**
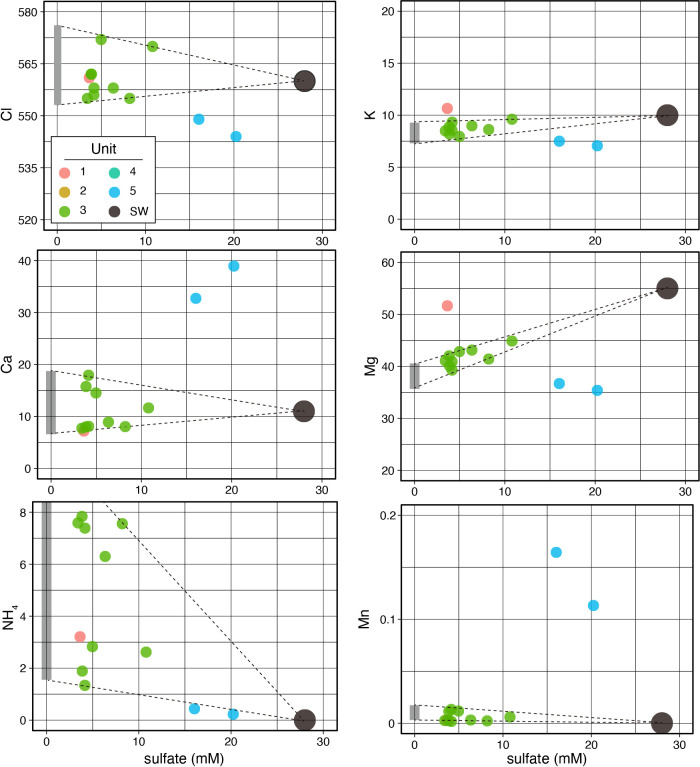
Pore-water chemistry. The ion concentrations of Cl, K, Ca, Mg, NH_4_, and Mn (a, b, c, d, e, and f) are plotted as a function of sulfate ion concentrations. Symbol colors represent the lithological units of samples. Broken lines connecting the seawater composition with the highest and lowest concentrations in Unit 3 samples are shown to indicate estimated endmember concentrations by the extrapolation of sulfate to zero, drawn by vertical grey bars.

Fig. 2 shows the chemical composition of pore-water samples. Fluid compositions were generally similar within Unit 1–3 samples, but differed between Unit 1–3 and Unit 5 samples. By assuming that all the sulfate detected in Unit 3 sample ana­lyses was derived from seawater contamination during sample treatments, the genuine composition of the other chemical components of Unit 3 pore-water samples may be estimated by the extrapolation of sulfate concentrations to zero. The estimated indigenous compositions of the selected ions of Unit 3 were similar to the seawater composition (Fig. 2). The same assumption of seawater contamination during sample recovery and treatment as that described above was applied to Unit 5 data; however, the estimated indigenous composition was not similar to the seawater composition. For example, the estimated indigenous concentrations of Ca and K (Fig. 2) were very high and low, respectively. Therefore, this assumption does not appear to be applicable to Unit 5 and the pore-water chemical composition elucidated for Unit 5 was not markedly affected by seawater contamination.

The pore-water of Unit 5 showed a quantitative change between Mg depletion and Ca enrichment as well as the slight loss of K and gain of Mn. The characteristics of the pore-water chemistry of Unit 5, higher Ca and lower Mg than the ambient seawater composition with a sufficient amount of sulfate, were consistent with those of the oceanic basement below sediment reported by other drilling projects (*e.g.* Elderfield *et al.*, 1999; Cowen *et al.*, 2003). These characteristics of oceanic basement water have been attributed to a low-temperature fluid-basalt interaction (*e.g.* Mottl and Holland, 1978; Mottl and Wheat, 1994). In a similar manner, the compositional and isotopic characteristics of sulfate in Unit 5 pore-water were affected by the precipitation of sulfate minerals, such as anhydrite, as well as diffusive sulfate loss to microbial sulfate reduction in the overlying prism sediments. Ammonium, the most probable product from the decomposition of sedimentary organic matter, was enriched in Unit 1–3 samples (>1‍ ‍mM), but not in Unit 5 samples.

The measured δD_H2O_ and δ^18^O_H2O_ values of pore-water were within a narrow range of between –0.02‰ and +3.82‰ and between –0.16‰ and +0.37‰, respectively (Table S1). Even if seawater (δD_H2O_ and δ^18^O_H2O_ of +0‰) significantly contaminated indigenous pore-water during sample recovery and processing (*e.g.*, up to 40% in volume based on sulfate concentrations. See Chester *et al.*, 2013a), variations in the δD_H2O_ value of indigenous pore-water were estimated to range between –0.1‰ and +6.4‰. Since this variation was markedly smaller than those in δD_H2_ and δD_CH4_ values described later, we assumed that the δD_H2O_ value of indigenous pore-water was +0‰ for later estimations of the isotopic fractionation effects of αD_CH4-H2O_ and αD_H2-H2O_ (see below).

### Carbon and hydrogen isotope systematics of CH_4_

**Fig. 3.**
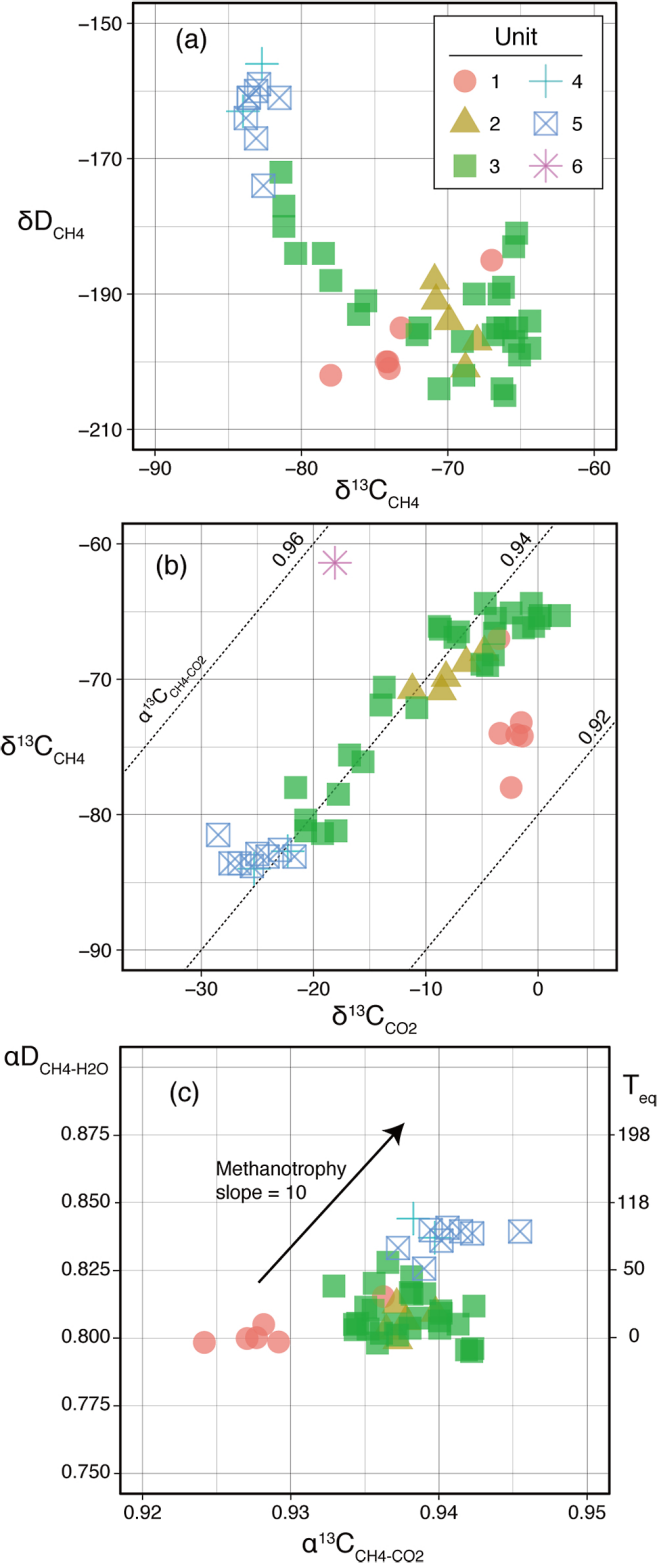
Methane isotope systematics. Each panel shows isotope composition plots between (a) δDCH_4_ and δ^13^CCH_4_, (b) δ^13^CCH_4_ and δ^13^CCO_2_, and (c) αDCH_4_-H_2_O and α^13^CCH_4_-CO_2_. Symbol colors and shapes represent the lithological units of samples. The diagonal dotted lines in panel (b) represent α^13^CCH_4_-CO_2_ values. The temperatures at which the isotope equilibrium between CH_4_ and H_2_O exhibited the corresponding αDCH_4_-H_2_O values are shown on the right side of the panel (c) (Turner *et al.*, 2021). The diagonal arrow in panel (c) represents the isotope effect associated with methanotrophy (αD/α^13^C=10).

CH_4_ concentrations varied between 1–20‍ ‍mM in Units 1–3, but were lower than 5‍ ‍mM from decollement to the depths of Units 4–6 (Fig. 1). Variations in CH_4_ concentrations were previously reported to be significantly affected by degassing during core recovery and processing (Chester *et al.*, 2013a); therefore, the measured concentrations may be underestimated from *in situ* concentrations. δ^13^C_CH4_ values were constant at approximately –64‰ in the upper half of Unit 3 and gradually decreased to –84‰ with increases in depth from the bottom half of Unit 3. δD_CH4_ values were almost constant at approximately –200‰ through Units 1–3 and increased along with a decrease in the δ^13^C_CH4_ value and increase in depth (Fig. 3a). δ^13^C_CO2_ values ranged between –‍30‰ and +0‰. Vertical changes in δ^13^C_CO2_ were similar to those in δ^13^C_CH4_. The vertical profiles of δ^13^C_CH4_ and δ^13^C_CO2_ values were continuous and smoother than that of CH_4_ concentrations (Fig. 1), suggesting the negligible effects of degassing during core recovery and processing on the isotopic composition.

Regarding the origin and behavior of CH_4_, isotope systematics between CH_4_ and the relevant C- and H-bearing molecules are shown in Fig. 3. The isotopic variation in CH_4_ appeared to be associated with variations in lithological units (Fig. 3a). As observed in vertical profiles, δ^13^C fractionation between CH_4_ and CO_2_ was constant at approximately 60‰ through Units 2–5 (Fig. 3b). It corresponded to an α^13^C_CH4-CO2_ value of approximately 0.94. The α^13^C_CH4-CO2_ value was the smallest at Unit 1 and the largest at Unit 6. α^13^C_CH4-CO2_ and αD_CH4-H2O_ values increased as the number of lithological units became higher (Fig. 3c).

### Stable isotope ratios of H_2_: observation and experiment

H_2_ concentrations in most of the gas samples analyzed (*n*=44 out of 52) were lower than 3.0‍ ‍μM (Fig. 1). In contrast, three serial samples at depths of 690–700 mbsf (4R-5R) showed H_2_ concentrations higher than 8‍ ‍μM, forming a sharp peak up to 209‍ ‍μM in the vertical profile (Fig. 1). Another H_2_ peak up to 26‍ ‍μM was detected at depths of 817–820 mbsf (15R-16R). Although extrinsic H_2_ generation during drilling and coring operations and sample processing is not completely excluded as the origin of the abundant H_2_ concentrations, all gas samples were processed by the same procedure and anomalies occurred among similar lithological samples (Unit 3). The amplitude of the increase in H_2_ from the background H_2_ level was significantly larger than that of variations in CH_4_ concentrations at the same depths because the concentrations of both of these gases are affected in a similar manner by the degassing process during core and sample recovery. Therefore, these H_2_ peaks may reflect indigenous H_2_ enrichment at specific depths.

**Fig. 4.**
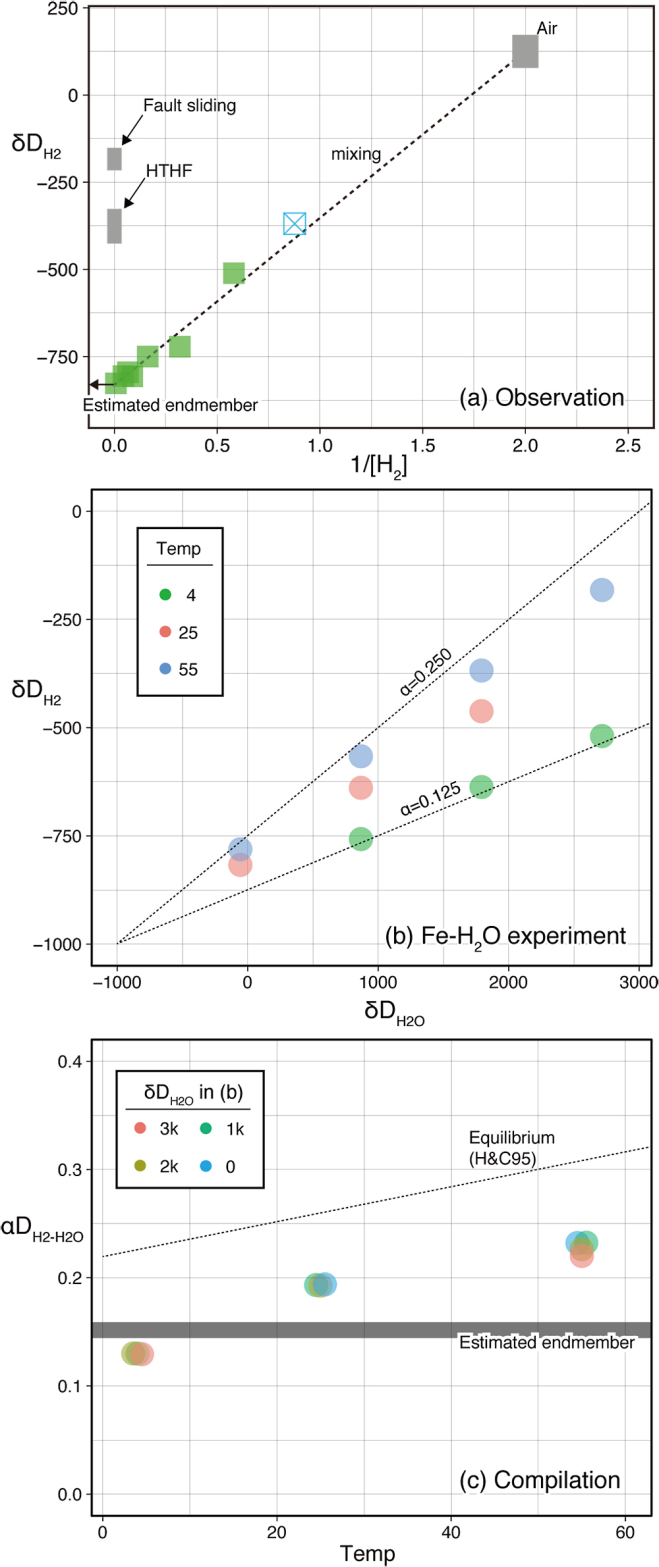
Molecular hydrogen isotope characteristics. Panel (a) shows a “Keeling plot” for the H_2_ observed. The symbols in panel (a) are the same as those in Fig. 3. The diagonal line represents the least square linear fit for the observation and atmospheric H_2_. Vertical grey bars represent isotope ratios reported for H_2_ generated by a fault sliding experiment (Hirose *et al.*, 2011) and observed in high-temperature hydrothermal fluid (HTHF) (Proskurowski *et al.*, 2006; Kawagucci *et al.*, 2010). Panel (b) shows the results of the Fe-H_2_O experiment in the present study. Each symbol represents a batch of experiments. Symbol colors represent reaction temperatures. Diagonal dotted lines represent the αD_H2-H2O_ values selected for comparison. Panel (c) is a compilation plot between the values resulting from the Fe-H_2_O experiment (the dataset is the same as that in panel (b)), αD_H2-H2O_ values and temperature for the estimated endmember value of the C0019E core observation, and the theoretical value at the isotope equilibrium between H_2_ and H_2_O (Horibe and Craig, 1995). Each symbol represents a batch of experiments. Symbol colors represent δD_H2O_ values at the experiment (3k means *ca.* +3000‰).

To discuss the possible source of the indigenous H_2_ enrichment observed, δD_H2_ values were quantified. The δD_H2_ values observed ranged between –850‰ and –350‰. The Keeling plot ana­lysis (Keeling, 1958), a plot between δD_H2_ values and the reciprocal of the H_2_ mixing ratio of the headspace gas, revealed a bimodal mixing trend between a D-rich low-H_2_ abundance endmember and a D-depleted H_2_-rich endmember with δD_H2_ of –850‰, corresponding to αD_H2-H2O_ of 0.15 (Fig. 4a). Since we recognized unavoidable contamination by H_2_ from air (0.5 ppmv with a δD_H2_ value of +150‰ [Gerst and Quay, 2000]) during head space gas sampling in the vial, the presence of a D-rich H_2_ endmember was reasonable. The single bimodal mixing trend and the estimated δD_H2_ value of approximately –850‰ for the H_2_-rich endmember through the samples suggested the following interpretations for the subseafloor H_2_ source: (1) on-site H_2_ generation occurring at multiple depths of the prism sediments, each of which showed an identical isotope effect, and/or (2) off-site H_2_ generation followed by the distribution of H_2_ to specific locations.

To address possible H_2_ generation and distribution processes in subseafloor environments, laboratory experiments were conducted to evaluate isotopic effects associated with H_2_ generation through a low-temperature Fe-H_2_O reaction. Experimentally generated H_2_ showed various δD_H2_ values, while clear correlations were observed between δD_H2_ and δD_H2O_ values at each reaction temperature (Fig. 4b). The αD_H2-H2O_ values of each batch experiment exhibited temperature-dependent correlations of 0.13, 0.19, and 0.23 at 4, 25, and 55°C, respectively (Fig. 4b and c). αD_H2-H2O_ values from the Fe-H_2_O experiment were lower than known αD_H2-H2O_ values at the H_2_-H_2_O isotope equilibrium at the corresponding temperatures, such as 0.23, 0.26, and 0.31 at 4, 25, and 55°C, respectively (Horibe and Craig, 1995). The αD_H2-H2O_ values of Fe-H_2_O experiments at 4–25°C were similar to that of the estimated D-depleted H_2_ endmember in the C0019E observation (Fig. 4a and c).

### Solid-phase biogeochemistry

**Fig. 5.**
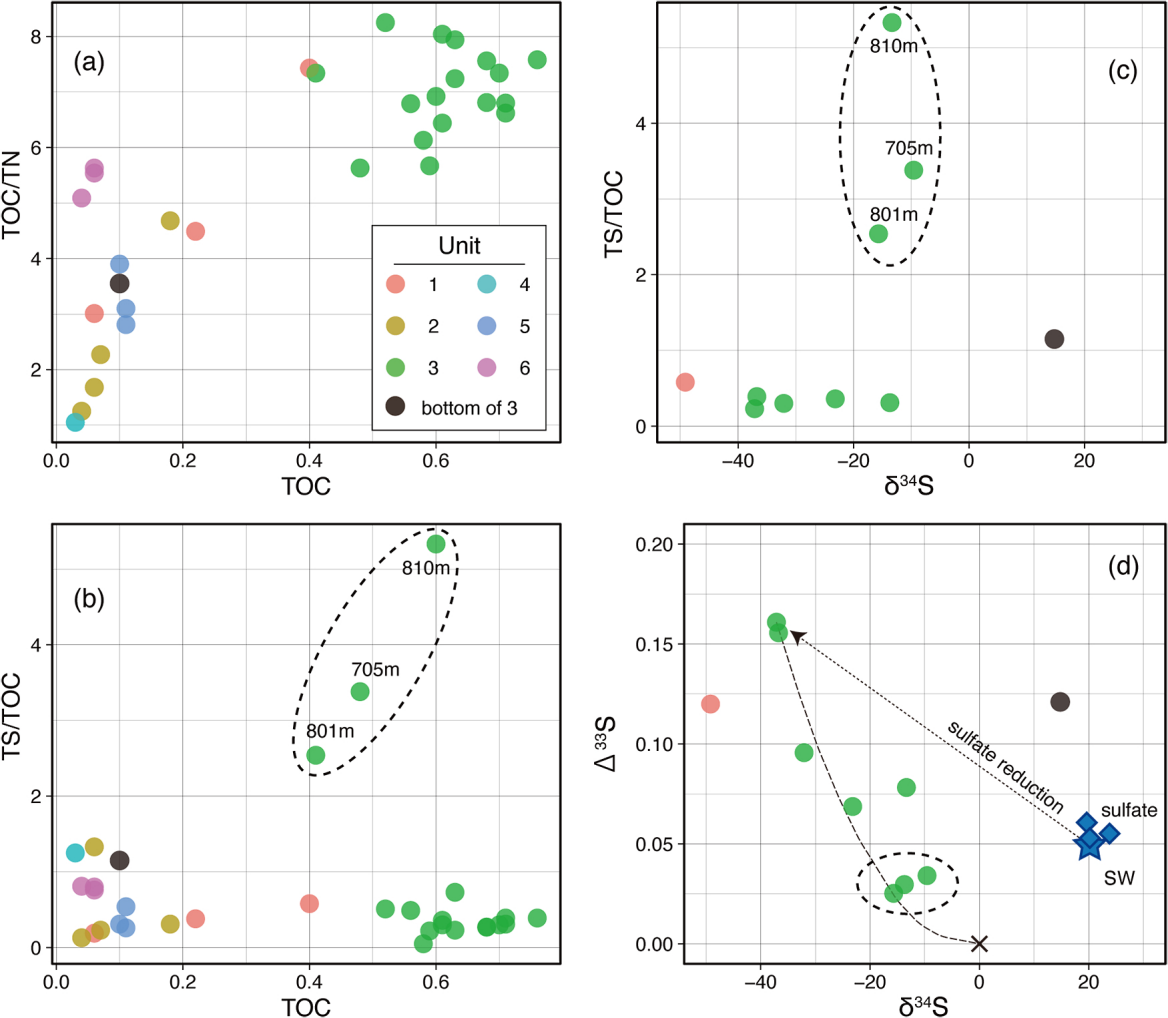
Solid-phase chemistry with sulfur isotope ratios. Panels (a) and (b) shows plots of the relative nitrogen content (TOC/TN) and relative sulfur content (TS/TOC) with the total organic carbon content (TOC). Panel (c) shows a plot between TS/TOC and the sulfur isotope ratios of CRS in squeezed cakes. Panel (d) shows three isotope plots for CRS as well as dissolved sulfate in pore-water (blue diamonds). A cross symbol represents the isotope composition of juvenile sulfur in the mantle. A dotted curve represents a theoretical mixing trend between juvenile sulfur and the sample with the highest Δ^33^S value. The arrow represents an isotope fractionation pattern associated with microbial sulfate reduction with 34ε of ~60 and 33λ of ~0.51 (see 3.4). Three samples with an anomalously high sulfur content are circled in panels (b-d). The filled circle in each panel represents a sample (15R-1) and its sulfur source is enigmatic.

Fig. 5 shows TOC, TN, and TS contents in the solid phase and the sulfur isotope composition of CRS. Core samples from units other than Unit 3 had a TOC content as low as 0.1% with TOC/TN ratios lower than 6. In contrast, samples from Unit 3 had a uniform TOC content of ~0.6% and a TOC/TN ratio of ~7 (Fig. 5a and Table S3). These characteristics are consistent with the terrigenous origin of Unit 3 introduced by the lithological description (Chester *et al.*, 2013a).

**Fig. 6.**
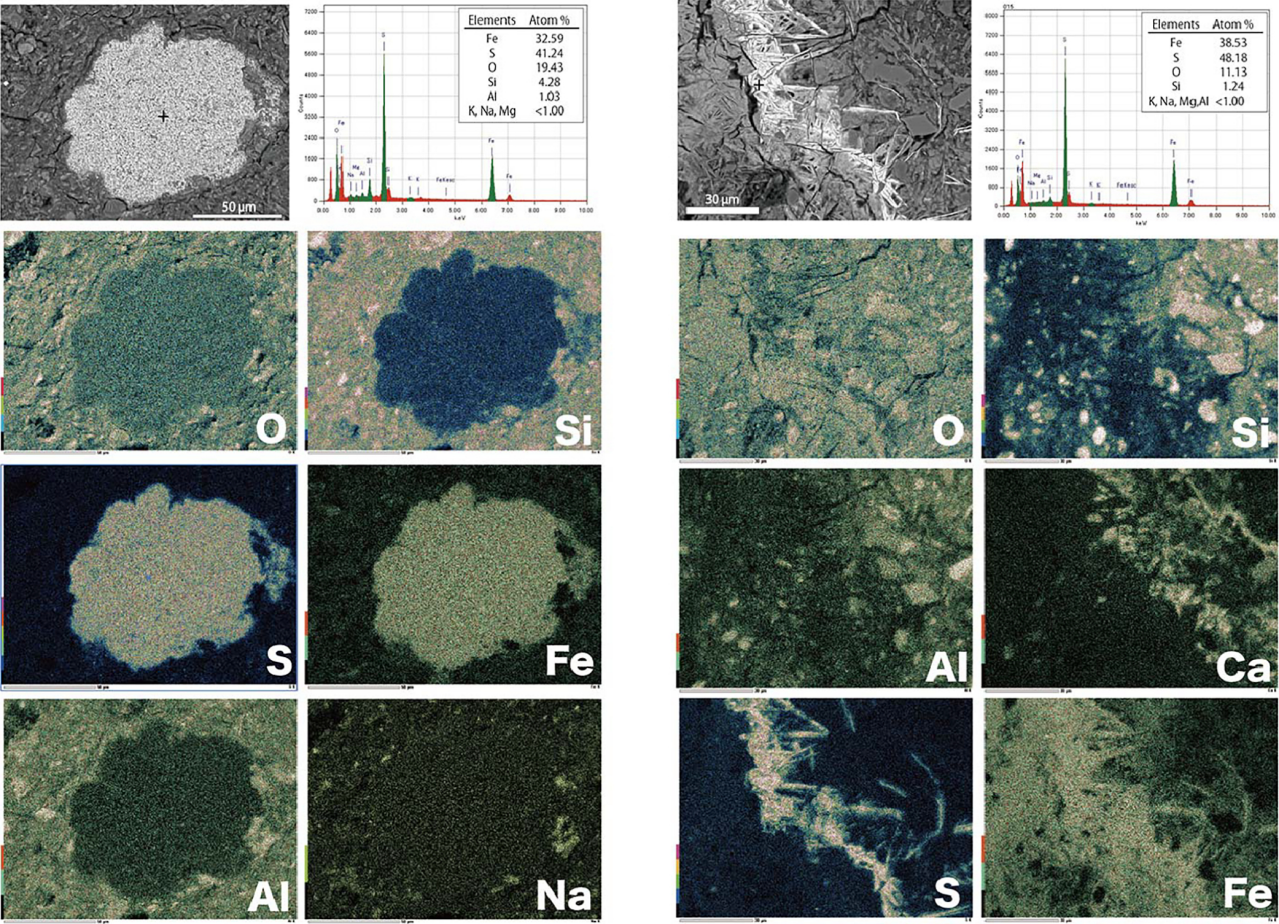
Two types of sulfide aggregates observed in Unit 3. (a) Framboidal aggregates and (b) an acicular aggregate with an EDS point ana­lysis and mapping. White/black images are back-scattered SEM images, and colored images are mapping results for each element. Each upper right spectrum is a point elemental ana­lysis at the point showing the black cross in each back-scattered image.

To trace the biogeochemical cycle of sulfur compounds in the deep subseafloor, the compositional, microscopic, and isotopic characteristics of solid-phase sulfur were examined. Relative sulfur contents (TS/TOC) were generally constant at approximately 0.5 through the depths examined regardless of TOC, and were >5-fold higher in samples collected at depths of ~705‍ ‍m (6R-2) and ~800–810‍ ‍m (13R-1 and 14R-1) (Fig. 5b). Locally high TS/TOC ratios were attributed to the occasional sampling of heterogeneously distributed sulfur-bearing aggregates, as suggested by the frequent occurrence of visible (>100‍ ‍μm) ellipsoidal to elongate aggregates in the dark mudstone of Unit 3 identified through onboard core imaging (Chester *et al.*, 2013a). Onshore SEM-EDS observations of thin sections from samples collected at depths of 715‍ ‍m (7R-2) and 802‍ ‍m (13R-2) revealed the distribution of iron-sulfide aggregates with framboidal or acicular structures (Fig. 6). Based on appearances and Fe/S stoichiometry, these aggregates were mainly pyrrhotite with minor pyrite. Similar sulfide aggregates were identified in the same core sections by another microscopic study (Yang *et al.*, 2018). Framboidal aggregates were scattered in the specimens, while acicular aggregates were only concentrated in discolored parts near the crack of core samples.

The δ^34^S values of CRS in the analyzed interval were markedly lower (–50 to –10‰) than those of pore-water sulfate (*ca.* +22‰) (Fig. 5c). Moreover, some Δ^33^S values of CRS (up to +0.16‰) were markedly higher than those of sulfate (Fig. 5d). It is important to note that the δ^34^S and Δ^33^S values of CRS in high TS/TOC samples were close to +0‰, corresponding to the values of juvenile sulfur from a mantle reservoir (*e.g.*, Ono *et al.*, 2012). Only one sample from 816.8‍ ‍m (15R-1) showed anomalous isotopic ratios with both positive δ^34^S and Δ^33^S values.

The apparent largest isotope fractionation between seawater sulfate and CRS, ^34^ε of 67.8‰ and ^33^λ of 0.514, was detected in Unit 1 (1R-1). This ^34^ε value was larger than the maximum ^34^ε value observed via microbial sulfate reduction in laboratory cultivations (Sim *et al.*, 2011) and was within the ^34^ε range in modern marine sediments (c.f., Wortmann *et al.*, 2001; Canfield *et al.*, 2010; Drake *et al.*, 2015). CRS with the lowest δ^34^S value in Unit 3 and seawater sulfate had ^34^ε and ^33^λ values of 55.3‰ and 0.514, respectively.

### Microbial cellular biomass and taxonomic composition

Prior to descriptions of microbial cell density and 16S rRNA gene amplicon sequences, it is important to note that data were obtained from core samples with a low biomass and potent external seawater contamination, as discussed above for pore-water sulfate. Therefore, results may have been affected by contamination and the biases of external seawater microbial populations as well as analytical methods during core recovery, processing, and laboratory experiments. Possible contamination by drilling mud fluids in microbiological core subsamples was measured onboard (Chester *et al.*, 2013a), and most of the core samples (except for 6R-2) used for microbial cell density and 16S rRNA gene amplicon sequencing ana­lyses were not markedly affected by contamination by drilling mud fluid (Chester *et al.*, 2013a).

**Fig. 7.**
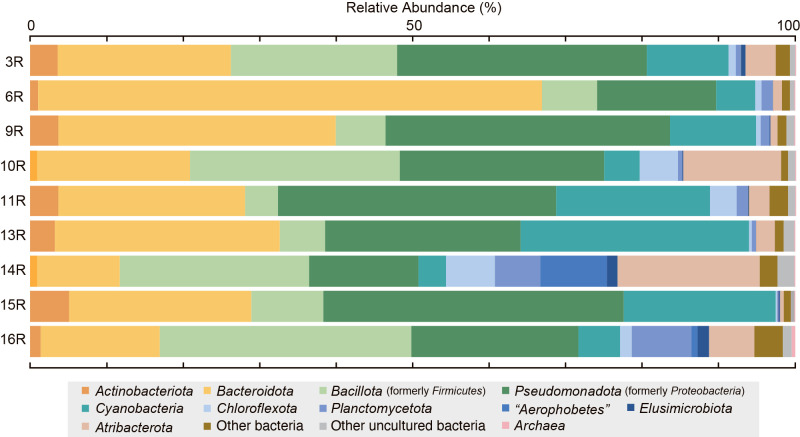
Summary of microbiological ana­lyses. Taxonomic composition of 16S rRNA gene amplicon sequences in sediments at site C0019E. Phylum-level taxonomic composition based on SSU rRNA gene amplicon sequencing using universal primers.

Microbial cell densities in core samples ranged between 4.4×10^4^ and 6.3×10^5^‍ ‍cells‍ ‍mL^–1^ of sediment (Table S2). According to the global biomass distribution in subseafloor sedimentary environments, a similar cell density was detected in sediments collected at similar depths below the subseafloor (Magnabosco *et al.*, 2018). The taxonomic compositions of core samples are shown in Fig. 7 and Fig. S2. The abundance of each phylogenetic group differed between lithological units; however, predominant members at the phylum level were common throughout the units; within *Bacteroidota* (11–66%), *Pseudomonadota* formerly named *Proteobacteria* (14–39%), *Bacillota* formerly named *Firmicutes* (4.2–32.3%), and *Cyanobacteria* (4–20%). In addition, *Flavobacteriaceae* members dominated within *Bacteroidota*, (80.7–96.9%) and accounted for approximately 60–70%, except 6R at 96%, in which the marine clusters NS2b, NS4, and NS5 and the genera *Tenacibaculum*, *Formosa*, *Cloacibacterium*, and *Aurantivirga* were core members. *Gammaproteobacteria* (5–18%) and *Alphaproteobacteria* (5–20%) members were the predominant components within *Pseudomonadota*, *Oleispira*, and *Pseudoalteromonas* were the predominant members of *Gammaproteobacteria*, and SAR11 and *Rhodobacteraceae*, including the genera *Amylibacter*, *Ascidiaceihabitans*, and *Cognatiyoonia*, were the dominant population for *Alphaproteobacteria*. On the other hand, *Chloroflexota* (0.2–6.4%), *Atribacterota* (0.4–18.5%), *Planctomycetota* (0.3–7.8%), and “*Aerophobetes*” (0.01–8.7%), which were frequently found in anoxic subseafloor sediments (Inagaki *et al.*, 2006; Nunoura *et al.*, 2016; Hoshino *et al.*, 2020), were also detected as less predominant members than the above components, even though these members were very abundant populations in the core samples of 10R and 14R. *Bacteroidota*, *Pseudomonadota*, and *Cyanobacteria* are key members of planktonic microbial communities in global seawater environments (Hoshino *et al.*, 2020), similar to the aforementioned pore-water sulfate contamination by seawater sulfate; therefore, the OTUs within these bacteria may be attributed to the contamination of seawater samples during sample recovery and processing (see Materials and Methods). *Bacteroidota* and *Pseudomonadota* were previously reported as major taxonomic members within drilling fluids (Yanagawa *et al.*, 2013; Martino *et al.*, 2019), even though the onboard contamination ana­lysis indicated that contamination by drilling mud fluid was negligible. Members of *Pseudomonadota* and *Flavobacteriaceae* were also detected in the shallow sediments of the Japan Trench (Hiraoka *et al.*, 2020), *Bacillota*, *Bacteroidota*, and *Pseudomonadota* were predominantly found in the ultradeep sediments of the Japan Trench forearc basin, and members of *Chloroflexota* and *Atribacterota* were identified in the same environments (Inagaki *et al.*, 2015); therefore, 16S rRNA gene compositions in prism sediments may be affected by the biodiversity of indigenous microbial communities.

The physiological properties of microorganisms corresponding to most of the OTUs constructed remain unknown, and the *in situ* functions of these potentially indigenous microbial populations remain unclear. However, if most of the OTUs within *Bacteroidota* and *Pseudomonadota* came from core samples, the predominant OTUs were most likely derived from the microbial taxa represented by a limited number of cultivated heterotrophic members, and some of the members of *Atribacterota* and *Chlorofrexiota* (*e.g.* *Anaerolineae*, *Dehalococcoidia*) have recently been identified as anaerobic heterotrophs (Kaster *et al.*, 2014; Katayama *et al.*, 2020; Yang *et al.*, 2020). Most of the microbial populations in Japan Trench prism sediments represented by the 16S rRNA gene amplicon sequencing ana­lysis appeared to be associated with the decomposition of refractory organic matter. Anaerobic methane oxidation coupled with sulfate reduction were experimentally inferred from carbon and sulfur isotope ana­lyses, and a few populations of sulfate reducers within the families *Desulfobacteraceae* and *Desulfobulbaceae*, both of which are associated with methanotrophic archaea (Dekas *et al.*, 2016), were detected in 13R and 16R samples. The presence of methanogens was not indicated from amplicon sequences and additionally performed PCR amplifications using specific primers of the genes for *mcrA*.

### Metabolic potentials and cultivations

The metabolic potentials of chemolithoautotrophic populations were examined using ^14^C-labeled substrates and detectable activities were found in several core samples (Table S2). In incubations with ^14^C-labeled bicarbonate, active homoacetogenesis (^14^C-labeled CO_2_ to CH_3_COOH) was observed in 5R, 8R, and 14R samples, while hydrogenotrophic methanogenesis (^14^C-labeled CO_2_ to CH_4_) was not present in any samples. Methanogenesis from ^14^C-methylamine was detected in the 13R sample and the anaerobic oxidation of ^14^C-labeled CH_4_ to CO_2_ in 1R, 6R, and 13R samples.

Based on the results obtained from the metabolic activity assessment of the microbial community, cultivations for homoacetogens as well as hydrogenotrophic and methylotrophic methanogens were conducted. Successful cultivation was achieved in the homoacetogenic medium with the 1R, 5R, 6R, 7R, 8R, and 13R samples, and the dilution cultivation technique estimated the cultivable population densities of homoacetogens in indigenous core samples as between 9.2×10^2^ and 9.6×10^4^‍ ‍cells‍ ‍mL^–1^ of sediment (Table S2). In addition, a positive cultivation for the methylotrophic methanogen population was obtained from the 13R sample and the estimated cultivable population density was 9.6×10^4^‍ ‍cells‍ ‍mL^–1^ of sediment. Enriched cultures in the most diluted media were subject to dilution-to-extinction techniques and the 16S rRNA gene sequences of isolates were elucidated. The 16S rRNA gene sequences of isolates from the homoacetogenic medium showed >99.9% similarity with each other, and were closely related to *Acetobacterium carbinolicum* (99%). The methylotrophic methanogen isolate was closely related to *Methanolobus taylorii* (99%). Although *A. carbinolicum* was isolated from subseafloor sediments of the Nankai Trough (at a water depth of 4,791‍ ‍m and at 4.15 mbsf) (Toffin *et al.*, 2004) and shallow marine sediments (Purdy *et al.*, 2003), there has been no cultivation example of related strains from hadal subseafloor sediments. Although the 16S rRNA amplicon sequence ana­lysis did not detect the signatures of the genera *Acetobacterium* and *Methanolobus* through core samples, they may represent indigenous populations not detected by the amplicon sequence ana­lysis. In cultivation tests, no hydrogenotrophic methanogens were obtained from any core samples. The cultivation of anoxic methanotrophs with sulfate reduction was not performed.

### Thermodynamic calculation

**Fig. 8.**
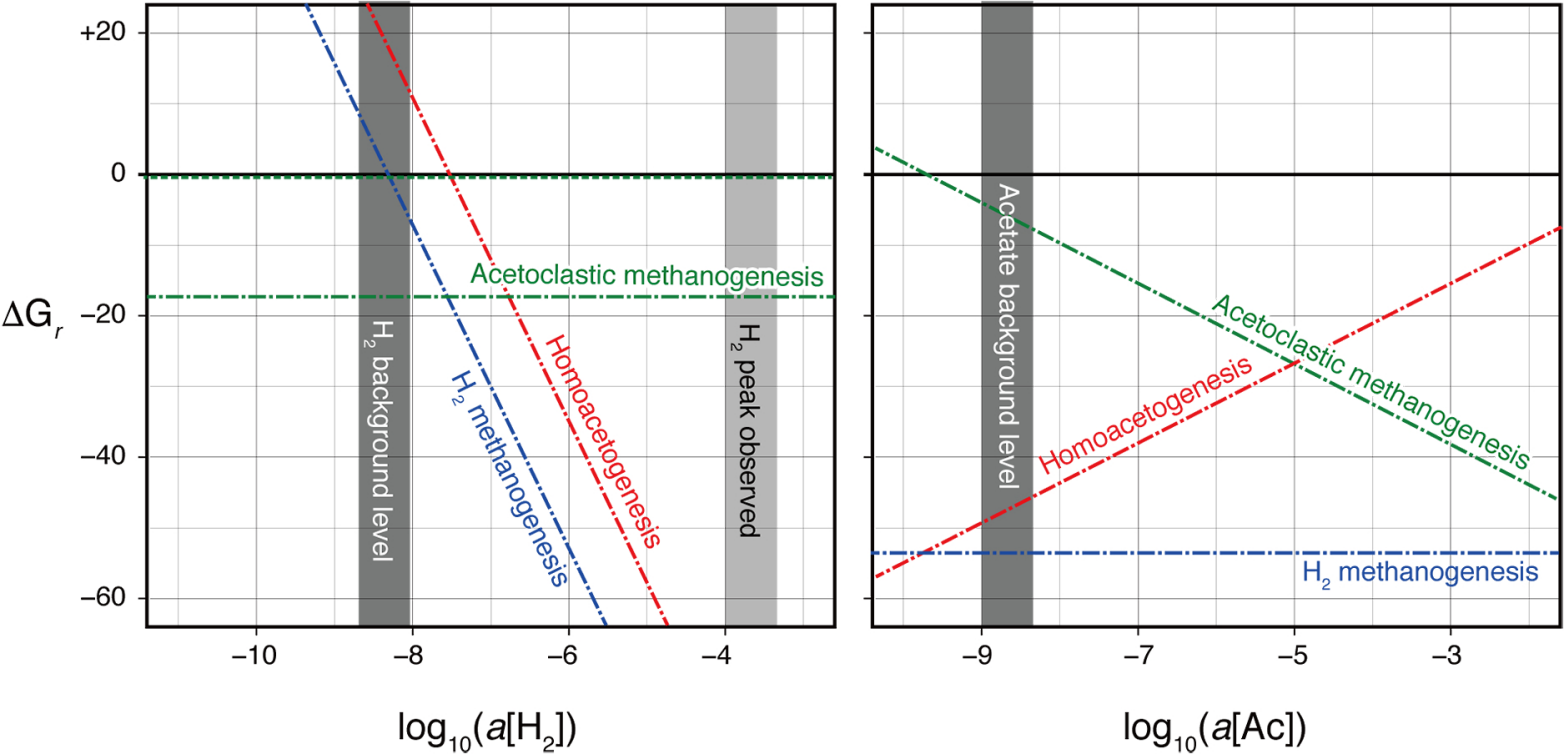
Gibbs free energy changes along with substrate activities for representative metabolism. Changes in Gibbs free energy changes with (a) *a*[H_2_] and (b) *a*[Acetate] for hydrogenotrophic methanogenesis, homoacetogenesis, and acetoclastic methanogenesis under the given conditions (pH=7.9, ΣCO_2_=50‍ ‍mM, and CH_4_=5‍ ‍mM) are shown. Horizontal dashed-dotted and dotted lines with dark and light green colors represent acetoclastic methanogenesis at ΣCH_3_COOH of 1 and 0.001‍ ‍mM, respectively.

The energetic potentials of hydrogenotrophic methanogenesis, homoacetogenesis, and aceticlastic methanogenesis were theoretically evaluated as *ΔG_r_* changes in response to changes in activities, *a*[H_2_] and *a*[Ac] values (Fig. 8). Homoacetogenesis and hydrogenotrophic methanogenesis were both exergonic at *a*[H_2_] as low as the detection limit of the H_2_ concentration used in the present study (~10^–6^ M), but were not exergonic under a possible H_2_ background level of ~10^–8^ M, quantified at other deep subseafloor areas with a more sensitive method (Lin *et al.*, 2012; Heuer *et al.*, 2020). When the concentration of acetate was constant as low as 1‍ ‍μM, corresponding to *a*[Ac] of 2.8×10^–10^, the *ΔG_r_* pattern with changes in *a*[H_2_] was similar between homoacetogenesis and hydrogenotrophic methanogenesis, while acetoclastic methanogenesis did not progress at any *a*[H_2_]. When the concentration of acetate was constant as high as 1‍ ‍mM, corresponding to *a*[Ac] of 2.8×10^–7^, the minimum threshold of *a*[H_2_] for homoacetogenesis (~10^–7^) was an order of magnitude higher than that of hydrogenotrophic methanogenesis (~10^–8^), while acetoclastic methanogenesis progressed at any *a*[H_2_]. The minimum threshold of *a*[Ac] for acetoclastic methanogenesis was ~10^9^, corresponding to an acetate concentration of ~10‍ ‍μM, as high as the background level of the acetate concentration in high-TOC sedimentary environments (Heuer *et al.*, 2009; Ijiri *et al.*, 2012).

## Discussion

### CH_4_ generation and maturation related to subseafloor microbial communities

CH_4_ was abundant in core samples of all accretionary prism sediments and appeared to be derived from microbial methanogenesis *in situ* and/or in proximal environments because of the *in situ* low-temperature condition (lower than 30°C) and the relatively low geothermal gradient of the Japan Trench accretionary wedge (Fulton *et al.*, 2013). The contribution of thermogenic hydrocarbons was previously suggested to be negligible due to the low temperature, which was reflected by the high methane/ethane ratios (~10^3^) observed (Chester *et al.*, 2013a).

CH_4_ generation and maturation related to subseafloor microbial communities in the prism sediment are hereafter discussed based on isotope systematics between CH_4_ and the relevant C- and H-bearing molecules. Low δ^13^C_CH4_ values (–‍84‰–64‰) and α^13^C_CH4-CO2_ values (0.92–0.94) (Fig. 3b) may both be attained through large kinetic isotope effects via hydrogenotrophic methanogenesis under the H_2_-limited condition (α^13^C_CH4-CO2_ of 0.91–0.94) (Valentine *et al.*, 2004; Penning *et al.*, 2005; Okumura *et al.*, 2016). Although the microbial population and function of hydrogenotrophic methanogens were not justified by cultivation-dependent or -independent ana­lyses in the present study, hydrogenotrophic methanogenesis associated with the fermentative decomposition of sedimentary organic matter may be operative in the accretionary wedge, which has frequently been suggested in similar subseafloor sedimentary environments (*e.g.* Inagaki *et al.*, 2015; Ijiri *et al.*, 2018; Beulig *et al.*, 2022).

Besides hydrogenotrophic methanogenesis, methylotrophic methanogenesis using methyl group carbons may also exert a large kinetic isotope effect (α^13^C_CH4-substrate_ of 0.93–0.95) (Krzycki *et al.*, 1987; Summons *et al.*, 1998). The activity of methylotrophic methanogenesis was detected in the core sample of 13R by both radioactive isotope tracer and cultivation tests (Fig. 7). Due to the terrigenous origin, the sediments of Unit 3 may host the typical metabolic products of coastal biota, such as trimethylamine N-oxide (TMAO) (Yancey *et al.*, 1982) and dimethylsulfoniopropionate (DMSP) (Kiene *et al.*, 2000), which may be substrates for methylotrophic methanogenesis. Therefore, methylotrophic methanogenesis may contribute to the abundance of biogenic CH_4_ in prism sediments as well as hydrogenotrophic methanogenesis.

The present study showed that the homoacetogenic population and its functions dominated the methanogenic population. The microbial processes initiated from homoacetogenesis may be potential sources of biogenic CH_4_. One example is aceticlastic methanogenesis directly using the end product (acetate) of homoacetogenesis, while another is hydrogenotrophic methanogenesis syntrophically coupled to acetate oxidation (to H_2_ and CO_2_) (Hattori, 2008), which may be catalyzed by different acetate-oxidizing populations from homoacetogens and even by the reverse acetogenesis reaction of homoacetogens. The apparent kinetic isotope effects of these methanogenic processes via homoacetogenesis are caused by the sequence of homoacetogenesis (α^13^C_Ac-CO2_=0.94) (Gelwicks *et al.*, 1989) and aceticlastic methanogenesis (α^13^C_CH4-Ac_=0.98–0.99) (Valentine *et al.*, 2004; Vinson *et al.*, 2017), which may have provided the low δ^13^C_CH4_ and α^13^C_CH4-CO2_ values observed.

While stable carbon isotopic signatures (*e.g.*, α^13^C_CH4-CO2_ values) provide an insight into the source of extant CH_4_, δD_CH4_ and αD_CH4-H2O_ values are associated with not only the source, but also the history of maturation. The constant αD_CH4-H2O_ values observed in the present study (~0.81) (Fig. 3c) suggest that extant CH_4_ in the prism sediments was not produced in recent years, it had accumulated through geological time. Biogenic CH_4_ has been shown to exhibit αD_CH4-H2O_ values of ~0.7 at its generation (*e.g.* Sugimoto and Wada, 1995). On the other hand, a compilation of αD_CH4-H2O_ values from various experiments and observations (Okumura *et al.*, 2016) revealed that αD_CH4-H2O_ values higher than 0.8 were the typical signatures of aged CH_4_ in geological samples regardless of their origin, as indicated by α^13^C_CH4-CO2_ values. The αD_CH4-H2O_ values of geological CH_4_ are consistent with the values of isotope equilibrium between CH_4_ and H_2_O at *in situ* temperatures (Gropp *et al.*, 2021; Turner *et al.*, 2021) (Fig. 3c). This was also the case for Units 1–3, for which αD_CH4-H2O_ thermometer values (0–50°C) (Fig. 3c) were consistent with the *in situ* temperature (<30°C) (Fulton *et al.*, 2013).

The slightly high δD_CH4_ values (>–180‰) found in the bottom of Unit 3 as well as Units 4–5 (Fig. 3a) appeared to be affected by microbial methane consumption, which enriches D and ^13^C with a δD/δ^13^C ratio of ~10 in remnant CH_4_ (Feisthauer *et al.*, 2011; Kawagucci *et al.*, 2021) (Fig. 3c). The metabolic function of anaerobic methane oxidation coupled with sulfate reduction was verified at several depths of the prism sediments by the radioactive isotope tracer assay (Table S2) despite this function not being detected from the boundary layer between Units 3 and 4. In addition, the vertical gradient of methane concentrations through the sediments between the bottom of Unit 3 and Units 4–5 (Fig. 1 and 2) indicated the occurrence and function of microbial methanotrophy.

### H_2_ generation and its distribution related to earthquakes and faults

In many oceanic and terrestrial subsurface sediments, H_2_ concentrations are generally lower than 0.1‍ ‍μM (Lin *et al.*, 2012). In the present study, H_2_ concentrations in most parts of C0019E core sediments were below the limit of quantification (<3‍ ‍μM) (Fig. 1). Unless local H_2_ inputs are present, a low H_2_ level is maintained most likely by subseafloor microbial functions, particularly by syntrophic microbial communities between hydrogenogenic fermenters and hydrogenotrophic chemolithotrophs. Therefore, kinetic properties for their utilization of H_2_ contribute to a steady-state H_2_ concentration (*e.g.* Lovley and Goodwin, 1988).

The sharp peaks observed in H_2_ concentrations at 690–700 and 817–820 mbsf in the present study (Fig. 1) demonstrated potential local H_2_ inputs. At approximately 700 mbsf, significant low gamma ray and resistivity signals were also identified by logging-while-drilling measurements (Chester *et al.*, 2013a). These findings suggest relationships between fault formation and local H_2_ inputs, leading to possible interpretations such as (1) on-site H_2_ generation around the faults and/or (2) remote H_2_ generation(s) and transportation via fault formation and fluid migration. Several processes have been reported for the abundant generation of H_2_ associated with fault activity: the water-rock redox reaction (Mayhew *et al.*, 2013), water radiolysis (Lin *et al.*, 2005), and mechanochemical water reduction associated with fault activity (Kita *et al.*, 1982; Hirose *et al.*, 2011) as well as the thermal decomposition of sedimentary organic matter (*e.g.* Kawagucci and Seewald, 2019).

Based on the field observations and possible H_2_ generation mechanisms described above, we hereafter discuss the source and distribution of H_2_ anomalies at specific depths using the hydrogen isotope ratio as a tracer. The highly D-depleted signature of H_2_ found at the peak of 700 mbsf (δD_H2_=–850‰ and αD_H2-H2O_=0.15) (Fig. 4a) strongly suggested that H_2_ was generated by a low-temperature process because of greater isotope effects (smaller αD_H2-H2O_) at lower temperatures. Based on the simulated experiments in the present study (Fig. 4b), a strong kinetic isotope effect (αD_H2-H2O_ value=0.15) was attainable by the generation of H_2_ via the low-temperature Fe-H_2_O reaction. Although zero valent iron used in the experiment was absent in the sedimentary environment, the strong kinetic isotope effect on H_2_ generation associated with the Fe-H_2_O reaction is of importance for the isotope effect associated with H_2_ generation through other low-temperature water-mineral interactions possibly occurring in the subseafloor, such as the Fe(II)-H_2_O interaction (Mayhew *et al.*, 2013).

In contrast, other processes for H_2_ expected around the fault zone cannot cause the D depletion in H_2_ observed. A simulated experiment of high-velocity fault sliding, resulting in the partial melting of rocks at the sliding surface, generated H_2_ with the D-rich signature of αD_H2-H2O_ of 0.8 (Hirose *et al.*, 2011) (Fig. 4a). High-temperature hydrothermal fluids in typical deep-sea hydrothermal vents contain H_2_, the isotope signatures of which are identical to the isotope equilibrium between H_2_ and H_2_O at *in situ* temperatures, namely, αD_H2-H2O_ of 0.6–0.7 at 300–400°C (*e.g.* Proskurowski *et al.*, 2006; Kawagucci *et al.*, 2010) (Fig. 4a). A previous thermal decomposition experiment on sedimentary organic matter also generated H_2_ isotopically equilibrated with H_2_O at given *in situ* high temperatures (Kawagucci and Seewald, 2019). Furthermore, microbial H_2_ metabolism, including its production and consumption, has been shown to promote the isotope equilibrium between environmental H_2_ and H_2_O (Walter *et al.*, 2012; Kawagucci *et al.*, 2014), while the H_2_-H_2_O isotope equilibrium results in a αD_H2-H2O_ value of not lower than 0.2 even at 0°C (Fig. 4c). These findings suggest that D-enriched H_2_ produced by high-temperature processes cannot be transformed to highly D-depleted H_2_ in the present study even through the later low-temperature isotopic equilibrium was mediated by the microbial metabolism of H_2_. This is one reason why we excluded the interpretation that remote H_2_ generation is associated with fault sliding and a subsequent hydrothermal reaction followed by H_2_ transportation via fault formation and fluid migration. Nevertheless, remote hot H_2_ generation followed by the subsequent stimulation of hydrogenotrophic activity that had previously occurred and had since ceased may be associated with possible geochemical and microbiological processes, such as methanogenesis and homoacetogenesis, other than the specific peaks of D-depleted H_2_.

**Fig. 9.**
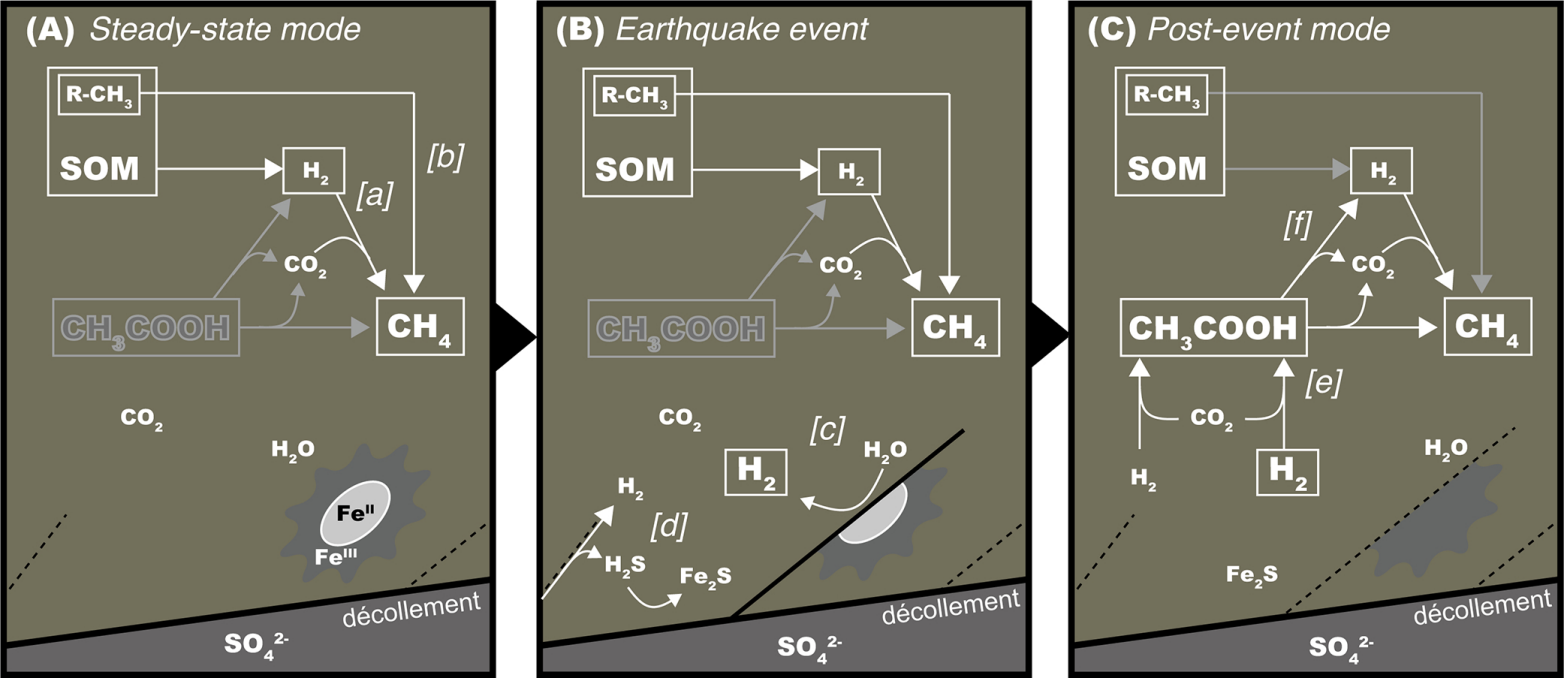
Illustration for the hypothesis. A hypothesis for microbial ecosystem dynamics at C0019E is shown. Panel A: The steady-state mode. [a] A syntrophic relationship between H_2_-generating fermentation and hydrogenotrophic methanogenesis as well as [b] methylotrophic methanogenesis continue to the accumulation of CH_4_. Panel B: Earthquake event. [c] On-site H_2_ generation associated with a low-temperature fluid-rock interaction and [d] H_2_S and H_2_ accompanied by coseismic hydrothermal fluid upwelling. Panel C: [e] homoacetogenesis and [f] accumulated acetate-based acetoclastic methanogenesis and reverse acetogenesis followed by hydrogenotrophic methanogenesis.

H_2_ generation through a low-temperature water-rock interaction may progress in natural fault zones. The significant generation of H_2_ was confirmed in simulated experiments between spinel-bearing rocks and water at 55°C within 10 days (Mayhew *et al.*, 2013). H_2_ generation was previously attributed to electron transfer from Fe(II) to H_2_O adsorbed on fresh spinel surfaces (Mayhew *et al.*, 2013). Since the rock surface in the subseafloor sedimentary lithosphere has been virtually passivated by changes through geological time, the Fe(II)-H_2_O interaction was generally not expected (Fig. 9A). On the other hand, even after these changes, sedimentary rock fragments still possessed an intact mineral composition in the inner part surrounded by the changed skin. Earthquake rupture exposes intact minerals to the rock surface and this is followed by subsequent interactions between the new surface and pore-water (Fig. 9B). Therefore, we speculate that relatively recent earthquakes (aftershocks after the 2011 Tohoku-oki earthquake) may have induced fault rupture at a depth of 700 mbsf at the drilling site and induced on-site H_2_ generation around the faults through the low-temperature water-rock redox interaction.

### Generation and distribution of solid-phase sulfur related to coseismic hydrothermal fluid flows

Processes associated with fault activity, suggested by carbon and hydrogen biogeochemistry as well as microbial functions, were examined in more detail based on solid-phase sulfur observations. The very low δ^34^S values with positive Δ^33^S values for CRS observed in Units 1 and 3 strongly suggest the involvement of microbial sulfate reduction in CRS production (Fig. 5d). Microbial sulfate reduction commonly occurs in relatively shallow subseafloor sediments during the early diagenesis of sedimentary organic matter and/or the sink of upwelling (seeping) CH_4_ from deep subseafloor reservoirs. CRS in Units 1 and 3 had been generated under sulfate-available conditions at shallow depths in the past and was then further buried with time.

Sulfur isotope systematics and TS/TOC ratios (Fig. 5d) as well as the sporadic distribution of aggregates (Fig. 6) may be attributed to the combination of juvenile sulfur transported by coseismic hydrothermal fluids (Yang *et al.*, 2018) and biogenic sulfide. Deep earthquake-induced processes near seismic centers, such as the frictional heating of basalt and peridotite, leads to a high-temperature fluid-rock interaction that forms coseismic hydrothermal fluids containing H_2_S with the juvenile sulfur isotope signature. Coseismic hydrothermal fluids then migrate through permeable fractures around the fault zones, followed by cooling, resulting in the local precipitation of sulfide minerals. Yang *et al.* (2018) indicated that framboidal pyrite/pyrrhotite aggregates formed when coseismic hydrothermal fluids encountered previously formed framboidal pyrite, while acicular pyrite/pyrrhotite aggregates formed when coseismic hydrothermal fluid leached the pre-existing pyrite and cooled within the fractures (Fig. 6). Although the effluent of coseismic hydrothermal fluids with deep-sourced CH_4_ and ^3^He-rich helium was captured around the epicenter 36 days after the 2011 Tohoku-oki Earthquake (Kawagucci *et al.*, 2012; Sano *et al.*, 2014), it remains unclear whether the sulfides observed in the present study originated from historical or recent earthquakes.

The presence of sulfides derived from coseismic hydrothermal fluid is consistent with the absence of hydrothermal fluid-derived H_2_ observed in the core samples as described above. Although seafloor high-temperature hydrothermal fluids contain both H_2_ and H_2_S (German and Seyfried, 2014; Nakamura and Takai, 2014), hydrothermal fluid-derived H_2_ is rapidly consumed by microbial metabolism without the precipitation of minerals. Therefore, any trace (even the D-enriched isotopic signature of high-temperature H_2_ generation) of hydrothermal fluid-derived H_2_ is expected to disappear, in contrast to H_2_S and sulfide minerals.

### Subseafloor microbial populations and functions

Subseafloor microbial communities in Japan Trench accretionary prism sediments without H_2_ enrichment may be sustained by sedimentary organic matter, which fuels the syntrophic populations of hydrogenogenic fermenters and hydrogenotrophic microbes, including methanogens (*e.g.* Lovley and Goodwin, 1988; Imachi *et al.*, 2011) (Fig. 9A). This sedimentary organic matter-based community, which may consist of *Bacteroidota*, *Pseudomonadota*, *Chloroflexota*, *Atribacterota*, and *Planctomycetota*, has also been identified in other deep subseafloor biospheres at the forearc basin of the Japan Trench and the accretionary prism of the Nankai Trough (Inagaki *et al.*, 2015; Nunoura *et al.*, 2016; Ijiri *et al.*, 2018; Beulig *et al.*, 2022). In addition, the population and metabolic functions of the methylotrophic methanogens detected in both the tracer activity ana­lysis and cultivation tests may be representative of the steady-state ecosystem in prism sediments (Fig. 9A). These steady-state communities and functions may result in the accumulation of CH_4_ at very slow rates through geological time since the oxidants capable of exothermically metabolizing CH_4_, such as sulfate, are not sufficient due to the distance from the seafloor and decollement.

The predominant potential of homoacetogenesis, not hydrogenotrophic methanogenesis, in the accretionary prism sediments under H_2_-rich conditions (Fig. 9C) was demonstrated by both radioactive isotope tracer ana­lyses and cultivation tests, while both shared the same substrates, H_2_ and CO_2_. Since the thermodynamic evaluation revealed that homoacetogenesis and hydrogenotrophic methanogenesis are energetically available under the given conditions of the laboratory experiments as well as the *in situ* H_2_-rich sediments (Fig. 8), the out-competition of homoacetogenesis may be attributed to differences in the kinetic nature between these hydrogeneotrophic chemolithtrophic populations under H_2_-rich conditions. The successful cultivation of homoacetogens was achieved by the medium with the 200-kPa headspace gas of 80% H_2_ (an order of 10^5^ Pa-H_2_ or 10^3^ μM H_2_). Significant acetogenic activities based on the radioactive isotope tracer assay were also obtained from media with the 100-kPa headspace gas of 1% H_2_ (an order of 10^3^ Pa-H_2_ or 10^1^ μM-H_2_). To date, the kinetic out-competition of homoacetogenesis under these H_2_-enriched conditions at low temperatures has been verified in laboratory cultivation experiments using several homoacetogen and methanogen isolates (Kotsyurbenko *et al.*, 2001).

At the time of the drilling expedition 1 year after the 2011 Tohoku-oki earthquake, we found H_2_ enrichment (up to an order of 10^4^ Pa-H_2_) and the significant potential of homoacetogenesis-predominant subseafloor microbial communities and functions (Fig. 9A). It remains unclear whether the tempo and mode in microbial communities and functions rapidly change in short periods or gradually in a geological time scale. However, from a local scale point of view, earthquake-induced H_2_ enrichment may be rapidly consumed by the response and enhancement of home-acetogenic populations and functions. Even after H_2_ consumption by homoacetogenic populations, the reduced concentrations of H_2_ at the minimum threshold for acetogens may still be available for other hydrogenotrophic populations. The theoretical calculation of metabolic thermo­dynamics indicates the exothermicity of hydrogenotrophic methanogenesis at lower concentrations of H_2_ than the thermo­dynamic minimum threshold for acetogens (Fig. 8). Labo­ratory cultivation experiments (Kotsyurbenko *et al.*, 2001) reported that the minimum threshold for H_2_ uptake was approximately 10^1^ Pa-H_2_ for acetogens and <10^0^ Pa-H_2_ for‍ ‍methanogens. In addition, a number of experiments using natural sediments demonstrated that the minimum threshold for mesophilic hydrogenotrophy was 10^–1^–10^0^ Pa-H_2_ (*e.g.* Lovley and Goodwin, 1988). Although the hydrogenotrophic metabolic activity in accretionary prism sediments at this low H_2_ level was not examined in the present study, multiple lines of evidence support the potential occurrence of hydrogenotrophy other than homoacetogenesis under the low H_2_ condition in the far-post-earthquake/pre-earthquake event era as a part of the steady-state ‘normal’ ecosystem.

### Conclusions and future perspectives

The geochemical and microbiological characteristics of the accretionary prism sediments of the Japan Trench 1 year after the 2011 Tohoku-oki earthquake were investigated. The 2011 Tohoku-oki earthquake was a type of millennial mega-earthquake occurring in similar segmentations of Japan Trench seismogenic zones (Ikehara *et al.*, 2016; Kodaira *et al.*, 2021). However, the geochemical and microbiological processes discussed in the present study were more frequent, not millennial, events because they progressed in a local scale and earthquake events follow the Gutenberg and Richter relationship (lower magnitude earthquakes occurring at a higher frequency). It is important to note that the sequence of seismicity, fluid-rock interactions, and microbial responses may occur anywhere in seismically-active subseafloors, not only in the Japan Trench. Nevertheless, since the primary chemical force is highly mobile and metabolizable H_2_, post-earthquake biogeochemical processes and microbial populations may be episodic and rapidly return to the steady-state ‘normal’ ecosystem (Fig. 9A) and, thus, may be difficult to detect even by scientific drilling operations, except in expeditions just after a mega-earthquake.

When an earthquake occurs in deep seismogenic zones and fault formation and sliding are activated and widespread, coseismic hydrothermal fluids are generated by frictional heating and migrate to accretionary prism sediments along the faults, which brings deep sources of H_2_ and H_2_S followed by sulfide precipitation due to the conductive cooling of the fluid (Fig. 9B). At accretionary prism sediments, the fresh surface of crushed rock around the faults interacts with pore-water, which serves as on-site H_2_ generation until the surface is completely changed. Earthquake-induced H_2_ inputs episodically drive the development of homoacetogenesis-predominant subseafloor microbial communities (Fig. 9C). When *in situ* H_2_ concentrations are below the minimum threshold for homoacetogenic metabolism, other hydrogenotrophic populations, such as methanogens, further consume H_2_ until their minimum threshold. Acetate, which accumulates as a result of temporal homoacetogenesis at a high H_2_ level, is also finally decomposed at a low H_2_ level into CH_4_. Thermodynamic calculations (Fig. 8) suggest the potential of acetoclastic methanogenesis and/or the acetate-dependent H_2_-syntrophic metabolism of reverse homoacetogenesis coupled with hydrogenotrophic methanogenesis (Fig. 9C). When episodic acetate supplied by homoacetogenic metabolism is virtually consumed, subseafloor microbial communities return to the steady-state tempo and mode (Fig. 9A).

However, there is still a lack of direct evidence to justify the hypothesis proposed here. For example, we did not obtain any data on *in situ* acetate concentrations for a quantitative discussion about microbial acetate production and consumption. The scare and coarse intervals of core sampling for onshore microbiological studies do not allow us to clarify the possible spatial transition of the metagenomics-based community structure around the H_2_ anomaly. If larger volumes of core samples are available, more specific cutting-edge ana­lyses, such as clumped isotopes of hydrocarbons (Eiler *et al.*, 2014; Gilbert *et al.*, 2019; Taguchi *et al.*, 2020) as well as metatranscriptomic comparisons of microbial communities (Orsi *et al.*, 2013; Zinke *et al.*, 2017, 2019), may be performed to justify the hypothesis. Nevertheless, JFAST drilling and geochemical and microbiological ana­lyses of core samples provide insights for the first time into the hadal and seismically active deep subseafloor biosphere. The relationship between earthquakes and the deep subsurface ecosystem will be the focus of future scientific drilling projects.

## Citation

Kawagucci, S., Sakai, S., Tasumi, E., Hirai, M., Takaki, Y., Nunoura, T., et al. (2023) Deep Subseafloor Biogeochemical Processes and Microbial Populations Potentially Associated with the 2011 Tohoku-oki Earthquake at the Japan Trench Accretionary Wedge (IODP Expedition 343). *Microbes Environ ***38**: ME22108.

https://doi.org/10.1264/jsme2.ME22108

## Supplementary Material

Supplementary Material

## References

[B1] Amend, J.P., McCollom, T.M., Hentscher, M., and Bach, W. (2011) Catabolic and anabolic energy for chemolithoautotrophs in deep-sea hydrothermal systems hosted in different rock types. Geochim Cosmochim Acta 75: 5736–5748.

[B3] Beulig, F., Schubert, F., Adhikari, R.R., Glombitza, C., Heuer, V.B., Hinrichs, K.-U., et al. (2022) Rapid metabolism fosters microbial survival in the deep, hot subseafloor biosphere. Nat Commun 13: 312. 35078973 10.1038/s41467-021-27802-7 PMC8789916

[B5] Bolyen, E., Rideout, J.R., Dillon, M.R., Bokulich, N.A., Abnet, C.C., Al-Ghalith, G.A., et al. (2019) Reproducible, interactive, scalable and extensible microbiome data science using QIIME 2. Nat Biotechnol 37: 852–857. 31341288 10.1038/s41587-019-0209-9 PMC7015180

[B7] Canfield, D.E., Farquhar, J., and Zerkle, A.L. (2010) High isotope fractionations during sulfate reduction in a low-sulfate euxinic ocean analog. Geology 38: 415–418.

[B8] Chester, F.M., Mori, J., Eguchi, N., Toczko, S., and the Expedition 343/343T Scientists. (2013a) *Proceedings of the Integrated Ocean Drilling Program*, 343/343T. Tokyo: Integrated Ocean Drilling Program Management International, Inc. doi:10.2204/iodp.proc.343343T.102.2013

[B9] Chester, F.M., Rowe, C., Ujiie, K., Kirkpatrick, J., Regalla, C., Remitti, F., et al. (2013b) Structure and composition of the plate-boundary slip zone for the 2011 Tohoku-Oki earthquake. Science 342: 1208–1211. 24311682 10.1126/science.1243719

[B10] Cowen, J.P., Giovannoni, S.J., Kenig, F., Johnson, H.P., Butterfield, D., Rappé, M.S., et al. (2003) Fluids from aging ocean crust that support microbial life. Science 299: 120–123. 12511653 10.1126/science.1075653

[B11] Dekas, A.E., Connon, S.A., Chadwick, G.L., Trembath-Reichert, E., and Orphan, V.J. (2016) Activity and interactions of methane seep microorganisms assessed by parallel transcription and FISH-NanoSIMS ana­lyses. ISME J 10: 678–692. 26394007 10.1038/ismej.2015.145 PMC4817681

[B12] de Ronde, C.E.J., Humphris, S.E., Höfig, T.W., Brandl, P.A., Cai, L., Cai, Y., *et al.* (2019) Brothers Arc Flux. In *Proceedings of the Ocean Drilling Program, Scientific Results* 376 College Station, TX: Integrated Ocean Drilling Program Management International, Inc.

[B13] D’Hondt, S., Inagaki, F., Zarikian, C.A., Abrams, L.J., Dubois, N., Engelhardt, T., et al. (2015) Presence of oxygen and aerobic communities from sea floor to basement in deep-sea sediments. Nat Geosci 8: 299–304.

[B14] Drake, H., Tullborg, E-.L., Whitehouse, M., Sandberg, B., Blomfeldt, T., and Åström, M.E. (2015) Extreme fractionation and micro-scale variation of sulphur isotopes during bacterial sulphate reduction in deep groundwater systems. Geochim Cosmochim Acta 161: 1–18.

[B15] Eiler, J.M., Bergquist, B., Bourg, I., Cartigny, P., Farquhar, J., Gagnon, A., et al. (2014) Frontiers of stable isotope geoscience. Chem Geol 372: 119–143.

[B16] Elderfield, H., Wheat, C.G., Mottl, M.J., Monnin, C., and Spiro, B. (1999) Fluid and geochemical transport through oceanic crust: a transect across the eastern flank of the Juan de Fuca Ridge. Earth Planet Sci Lett 172: 151–165.

[B18] Feisthauer, S., Vogt, C., Modrzynski, J., Szlenkier, M., Krüger, M., Siegert, M., et al. (2011) Different types of methane monooxygenases produce similar carbon and hydrogen isotope fractionation patterns during methane oxidation. Geochim Cosmochim Acta 75: 1173–1184.

[B20] Früh-Green, G.L., Orcutt, B.N., Rouméjon, S., Lilley, M.D., Morono, Y., Cotterill, C., et al. (2018) Magmatism, serpentinization and life: Insights through drilling the Atlantis Massif (IODP Expedition 357). Lithos 323: 137–155.

[B21] Fulton, P.M., Brodsky, E.E., Kano, Y., Mori, J., Chester, F., Ishikawa, T., et al. (2013) Low coseismic friction on the Tohoku-Oki fault determined from temperature measurements. Science 342: 1214–1217. 24311684 10.1126/science.1243641

[B22] Gelwicks, J.T., Risatti, J.B., and Hayes J.M. (1989) Carbon isotope effects associated with autotrophic acetogenesis. Org Geochem 14: 441–446. 11542159 10.1016/0146-6380(89)90009-0

[B23] German, C.R., and Seyfried, W.E. (2014) 8.7-Hydrothermal Processes. In *Treatise on Geochemistry 2nd ed.* Holland H.D., and Turekian K.K. (eds). Amsterdam: Elsevier, pp. 191–233.

[B24] Gerst, S., and Quay, P. (2000) The deuterium content of atmospheric mole­cular hydrogen: Method and initial measurements. J Geophys Res: Atmos 105: 26433–26445.

[B25] Gieskes, J.M., Vrolijk, P., and Blanc, G. (1990) Hydrogeochemistry of the northern Barbados accretionary complex transect: Ocean Drilling Project leg 110. J Geophys Res: Solid Earth 95: 8809–8818.

[B26] Gieskes, J.M., Gamo, T., and Kastner, M. (1993) Major and minor element geochemistry of interstitial waters of site 808, Nankai trough: an overview. In *Proceedings of the Ocean Drilling Program, Scientific Results* 131: College Station, TX: Integrated Ocean Drilling Program Management International, Inc, pp.387–396.

[B27] Gilbert, A., Lollar, B.S., Musat, F., Giunta, T., Chen, S., Kajimoto, Y., et al. (2019) Intramole­cular isotopic evidence for bacterial oxidation of propane in subsurface natural gas reservoirs. Proc Natl Acad Sci U S A 116: 201817784. 10.1073/pnas.1817784116 PMC6452727 30886103

[B28] Gropp, J., Iron, M.A., and Halevy, I. (2021) Theoretical estimates of equilibrium carbon and hydrogen isotope effects in microbial methane production and anaerobic oxidation of methane. Geochim Cosmochim Acta 295: 237–264.

[B29] Hales, B.A., Edwards, C., Ritchie, D.A., Hall, G., Pickup, R.W., and Saunders, J.R. (1996) Isolation and identification of methanogen-specific DNA from blanket bog peat by PCR amplification and sequence ana­lysis. Appl Environ Microbiol 62: 668–675. 8593069 10.1128/aem.62.2.668-675.1996 PMC167834

[B125] Hattori, S. (2008) Syntrophic acetate-oxidizing microbes in methanogenic environments. Microbes Environ 23: 118–127. 21558697 10.1264/jsme2.23.118

[B30] Hensen, C., and Wallmann, K. (2005) Methane formation at Costa Rica continental margin—constraints for gas hydrate inventories and cross-décollement fluid flow. Earth Planet Sci Lett 236: 41–60.

[B31] Heuer, V.B., Pohlman, J.W., Torres, M.E., Elvert, M., and Hinrichs, K.-U. (2009) The stable carbon isotope biogeochemistry of acetate and other dissolved carbon species in deep subseafloor sediments at the northern Cascadia Margin. Geochim Cosmochim Acta 73: 3323–3336.

[B32] Heuer, V.B., Inagaki, F., Morono, Y., Kubo, Y., Spivack, A.J., Viehweger, B., et al. (2020) Temperature limits to deep subseafloor life in the Nankai Trough subduction zone. Science 370: 1230–1234. 33273103 10.1126/science.abd7934

[B33] Hiraoka, S., Hirai, M., Matsui, Y., Makabe, A., Minegishi, H., Tsuda, M., et al. (2020) Microbial community and geochemical ana­lyses of trans-trench sediments for understanding the roles of hadal environments. ISME J 14: 740–756. 31827245 10.1038/s41396-019-0564-z PMC7031335

[B34] Hirose, T., Kawagucci, S., and Suzuki, K. (2011) Mechanoradical H_2_ generation during simulated faulting: Implications for an earthquake-driven subsurface biosphere. Geophys Res Lett 38: L17303.

[B35] Horibe, Y., and Craig, H. (1995) D/H fractionation in the system methane-hydrogen-water. Geochim Cosmochim Acta 59: 5209–5217.

[B36] Hoshino, T., Doi, H., Uramoto, G.-I., Wörmer, L., Adhikari, R.R., Xiao, N., et al. (2020) Global diversity of microbial communities in marine sediment. Proc Natl Acad Sci U S A 117: 27587–27597. 33077589 10.1073/pnas.1919139117 PMC7959581

[B37] Hsieh, Y.P., and Shieh, Y.N. (1997) Analysis of reduced inorganic sulfur by diffusion methods: improved apparatus and evaluation for sulfur isotopic studies. Chem Geol 137: 255–261.

[B38] Ijiri, A., Harada, N., Hirota, A., Tsunogai, U., Ogawa, N.O., Itaki, T., et al. (2012) Biogeochemical processes involving acetate in sub-seafloor sediments from the Bering Sea shelf break. Org Geochem 48: 47–55.

[B39] Ijiri, A., Inagaki, F., Kubo, Y., Adhikari, R.R., Hattori, S., Hoshino, T., et al. (2018) Deep-biosphere methane production stimulated by geofluids in the Nankai accretionary complex. Sci Adv 4: eaao4631. 29928689 10.1126/sciadv.aao4631 PMC6007163

[B40] Ikehara, K., Kanamatsu, T., Nagahashi, Y., Strasser, M., Fink, H., Usami, K., et al. (2016) Documenting large earthquakes similar to the 2011 Tohoku-oki earthquake from sediments deposited in the Japan Trench over the past 1500 years. Earth Planet Sci Lett 445: 48–56.

[B41] Imachi, H., Aoi, K., Tasumi, E., Saito, Y., Yamanaka, Y., Saito, Y., et al. (2011) Cultivation of methanogenic community from subseafloor sediments using a continuous-flow bioreactor. ISME J 5: 1913–1925. 21654849 10.1038/ismej.2011.64 PMC3223304

[B43] Inagaki, F., Nunoura, T., Nakagawa, S., Teske, A., Lever, M., Lauer, A., et al. (2006) Biogeographical distribution and diversity of microbes in methane hydrate-bearing deep marine sediments on the Pacific Ocean Margin. Proc Natl Acad Sci U S A 103: 2815–2820. 16477011 10.1073/pnas.0511033103 PMC1413818

[B44] Inagaki, F., Hinrichs, K.U., Kubo, Y., Bowles, M.W., Heuer, V.B., Hong, W.L., et al. (2015) Exploring deep microbial life in coal-bearing sediment down to 2.5‍ ‍km below the ocean floor. Science 349: 420–424. 26206933 10.1126/science.aaa6882

[B45] Johnson, J.W., Oelkers, E.H., and Helgeson, H.C. (1992) SUPCRT92: a software package for calculating the standard molal thermodynamic properties of minerals, gases, aqueous species, and reactions from 1 to 5000 bar and 0 to 1000°C. Comput Geosci 18: 899–947.

[B46] Kallmeyer, J., Pockalny, R., Adhikari, R.R., Smith, D.C., and D’Hondt, S. (2012) Global distribution of microbial abundance and biomass in subseafloor sediment. Proc Natl Acad Sci U S A 109: 16213–16216. 22927371 10.1073/pnas.1203849109 PMC3479597

[B47] Kaster, A.K., Mayer-Blackwell, K., Pasarelli, B., and Spormann, A.M. (2014) Single cell genomic study of *Dehalococcoidetes* species from deep-sea sediments of the Peruvian Margin. ISME J 8:1831–1842. 24599070 10.1038/ismej.2014.24 PMC4139717

[B48] Katayama, T., Nobu, M.K., Kusada, H., Meng, X.-Y., Hosogi, N., Uematsu, K., et al. (2020) Isolation of a member of the candidate phylum “Atribacteria” reveals a unique cell membrane structure. Nat Commun 11: 1–9. 33318506 10.1038/s41467-020-20149-5 PMC7736352

[B49] Kawagucci, S., Toki, T., Ishibashi, J., Takai, K., Ito, M., Oomori, T., et al. (2010) Isotopic variation of mole­cular hydrogen in 20°–375°C hydrothermal fluids as detected by a new analytical method. J Geophys Res Biogeosci 115: G03021-9.

[B50] Kawagucci, S., Yoshida, Y.T., Noguchi, T., Honda, M.C., Uchida, H., Ishibashi, H., et al. (2012) Disturbance of deep-sea environments induced by the M9.0 Tohoku Earthquake. Sci Rep 2: 270. 22355782 10.1038/srep00270 PMC3280601

[B51] Kawagucci, S., Kobayashi, M., Hattori, S., Yamada, K., Ueno, Y., Takai, K., et al. (2014) Hydrogen isotope systematics among H_2_-H_2_O-CH_4_ during the growth of the hydrogenotrophic methanogen *Methanothermobacter thermautotrophicus* strain ΔH. Geochim Cosmochim Acta 142: 601–614.

[B52] Kawagucci, S., Miyazaki, J., Morono, Y., Seewald, J.S., Wheat, C.G., and Takai, K. (2018) Cool, alkaline serpentinite formation fluid regime with scarce microbial habitability and possible abiotic synthesis beneath the South Chamorro Seamount. Prog Earth Planet Sci 5: 74.

[B53] Kawagucci, S., and Seewald, J.S. (2019) Compositional and isotopic characteristics of hydrocarbons generated by a hydrothermal experiment simulating seafloor sediment alteration stepwise heating from 275 to 361°C at 30 MPa. Geochem J 53: 281–291.

[B54] Kawagucci, S., Matsui, Y., Makabe, A., Fukuba, T., Onishi, Y., Nunoura, T., et al. (2021) Hydrogen and carbon isotope fractionation factors of aerobic methane oxidation in deep-sea water. Biogeosciences 18: 5351–5362.

[B55] Keeling, C.D. (1958) The concentration and isotopic abundances of atmospheric carbon dioxide in rural areas. Geochim Cosmochim Acta 13: 322–334.

[B56] Kiene, R.P., Linn, L.J., and Bruton, J.A. (2000) New and important roles for DMSP in marine microbial communities. J Sea Res 43: 209–224.

[B58] Kita, I., Matsuo, S., Wakita, H., and Nakamura, Y. (1980) D/H ratios of H_2_ in soil gases as an indicator of fault movements. Geochem J 14: 317–320.

[B59] Kita, I., Matsuo, S., and and Wakita, H. (1982) H_2_ generation by reaction between H_2_O and crushed rock: An experimental study on H_2_ degassing from the active fault zone. J Geophys Res 87: 10789–10795.

[B60] Kodaira, S., Iinuma, T., and Imai, K. (2021) Investigating a tsunamigenic megathrust earthquake in the Japan Trench. Science 371: eabe1169. 33707238 10.1126/science.abe1169

[B61] Kotsyurbenko, O.R., Glagolev, M.V., Nozhevnikova, A.N., and Conrad, R. (2001) Competition between homoacetogenic bacteria and methanogenic archaea for hydrogen at low temperature. FEMS Microbiol Ecol 38: 153–159.

[B62] Krzycki, J.A., Kenealy, W.R., Deniro, M.J., and Zeikus, J.G. (1987) Stable Carbon Isotope Fractionation by *Methanosarcina barkeri* during methanogenesis from acetate, methanol, or carbon dioxide-hydrogen. Appl Environ Microbiol 53: 2597–2599. 16347476 10.1128/aem.53.10.2597-2599.1987 PMC204153

[B63] Lever, M.A., Rouxel, O., Alt, J.C., Shimizu, N., Ono, S., Coggon, R.M., et al. (2013) Evidence for microbial carbon and sulfur cycling in deeply buried ridge flank basalt. Science 339: 1305–1308. 23493710 10.1126/science.1229240

[B64] Lever, M.A., and Teske, A.P. (2015) Diversity of methane-cycling archaea in hydrothermal sediment investigated by general and group-specific PCR primers. Appl Environ Microbiol 81: 1426–1441. 25527539 10.1128/AEM.03588-14 PMC4309701

[B127] Lin, L-H., Slater, G.F., Sherwood-Lollar, B., Lacrampe-Couloume, G., Onstott, T.C. (2005) The yield and isotopic composition of radiolytic H2, a potential energy source for the deep subsurface biosphere. Geochim Cosmochim Acta 69: 893–903.

[B65] Lin, Y.-S., Heuer, V.B., Goldhammer, T., Kellermann, M.Y., Zabel, M., and Hinrichs, K.-U. (2012) Towards constraining H_2_ concentration in subseafloor sediment: a proposal for combined ana­lysis by two distinct approaches. Geochim Cosmochim Acta 77: 186–201.

[B67] Lovley, D.R., and Goodwin, S. (1988) Hydrogen concentrations as an indicator of the predominant terminal electron-accepting reactions in aquatic sediments. Geochim Cosmochim Acta 52: 2993–3003.

[B68] Magnabosco, C., Lin, L.H., Dong, H., Bomberg, M., Ghiorse, W., Stan-Lotter, H., et al. (2018) The biomass and biodiversity of the continental subsurface. Nat Geosci 11: 707–717.

[B69] Martin, M. (2011) Cutadapt removes adapter sequences from high-throughput sequencing reads. EMBnet J 17: 10–12.

[B70] Martino, A., Rhodes, M.E., León-Zayas, R., Valente, I.E., Biddle, J.F., and House, C.H. (2019) Microbial diversity in sub-seafloor sediments from the Costa Rica margin. Geosciences (Basel, Switz) 9: 2076–3263.

[B71] Mayhew, L.E., Ellison, E.T., McCollom, T.M., Trainor, T.P., and Templeton, A.S. (2013) Hydrogen generation from low-temperature water-rock reactions. Nat Geosci 6: 478–484.

[B72] McCollom, T.M., and Shock, E.L. (1997) Geochemical constraints on chemolithoautotrophic metabolism by microorganisms in seafloor hydrothermal systems. Geochim Cosmochim Acta 61:4375–4391. 11541662 10.1016/s0016-7037(97)00241-x

[B74] Morono, Y., Terada, T., Nishizawa, M., Ito, M., Hillion, F., Takahata, N., et al. (2011) Carbon and nitrogen assimilation in deep subseafloor microbial cells. Proc Natl Acad Sci U S A 108: 18295–18300. 21987801 10.1073/pnas.1107763108 PMC3215001

[B75] Mottl, M.J., and Holland, H.D. (1978) Chemical exchange during hydrothermal alteration of basalt by seawater—I. Experimental results for major and minor components of seawater. Geochim Cosmochim Acta 42: 1103–1115.

[B76] Mottl, M.J., and Wheat, C.G. (1994) Hydrothermal circulation through mid-ocean ridge flanks: Fluxes of heat and magnesium. Geochim Cosmochim Acta 58: 2225–2237.

[B77] Mottl, M.J., Komor, S.C., Fryer, P., and Moyer, C.L. (2003) Deep-slab fluids fuel extremophilic Archaea on a Mariana forearc serpentinite mud volcano: Ocean Drilling Program Leg 195. Geochem, Geophys, Geosyst 4: 9009.

[B78] Nakamura, K., and Takai, K. (2014) Theoretical constraints of physical and chemical properties of hydrothermal fluids on variations in chemolithotrophic microbial communities in seafloor hydrothermal systems. Prog Earth Planet Sci 1: 5.

[B79] Nunoura, T., Oida, H., Miyazaki, J., Miyashita, A., Imachi, H., and Takai, K. (2008) Quantification of mcrA by fluorescent PCR in methanogenic and methanotrophic microbial communities. FEMS Microbiol Ecol 64: 240–247. 18318714 10.1111/j.1574-6941.2008.00451.x

[B80] Nunoura, T., Takaki, Y., Kazama, H., Hirai, M., Ashi, J., Imachi, H., et al. (2012) Microbial diversity in deep-sea methane seep sediments presented by SSU rRNA gene tag sequencing. Microbes Environ 27: 382–390. 22510646 10.1264/jsme2.ME12032 PMC4103545

[B81] Nunoura, T., Takaki, Y., Shimamura, S., Kakuta, J., Kazama, H., Hirai, M., et al. (2016) Variance and potential niche separation of microbial communities in subseafloor sediments off Shimokita Peninsula, Japan. Environ Microbiol 18: 1889–1906. 26486095 10.1111/1462-2920.13096

[B83] Okumura, T., Kawagucci, S., Saito, Y., Matsui, Y., Takai, K., and Imachi, H. (2016) Hydrogen and carbon isotope systematics in hydrogenotrophic methanogenesis under H_2_-limited and H_2_-enriched conditions: implications for the origin of methane and its isotopic diagnosis. Prog Earth Planet Sci 3: 219.

[B84] Ono, S., Keller, N.S., Rouxel, O., and Alt, J.C. (2012) Sulfur-33 constraints on the origin of secondary pyrite in altered oceanic basement. Geochim Cosmochim Acta 87: 323–340.

[B85] Orsi, W.D., Edgcomb, V.P., Christman, G.D., and Biddle, J.F. (2013) Gene expression in the deep biosphere. Nature 499: 205–208. 23760485 10.1038/nature12230

[B86] Penning, H., Plugge, C.M., Galand, P.E., and Conrad, R. (2005) Variation of carbon isotope fractionation in hydrogenotrophic methanogenic microbial cultures and environmental samples at different energy status. Global Change Biol 11: 2103–2113. 10.1111/j.1365-2486.2005.01076.x 34991282

[B87] Proskurowski, G., Lilley, M.D., Kelley, D.S., and Olson, E.J. (2006) Low temperature volatile production at the Lost City Hydrothermal Field, evidence from a hydrogen stable isotope geothermometer. Chem Geol 229: 331–343.

[B88] Purdy, K.J., Nedwell, D.B., and Embley, T.M. (2003) Analysis of the sulfate-reducing bacterial and methanogenic archaeal populations in contrasting Antarctic sediments. Appl Environ Microbiol 69: 3181–3191. 12788715 10.1128/AEM.69.6.3181-3191.2003 PMC161550

[B89] Quast, C., Pruesse, E., Yilmaz, P., Gerken, J., Schweer, T., Yarza, P., et al. (2013) The SILVA ribosomal RNA gene database project: improved data processing and web-based tools. Nucleic Acids Res 41: D590–D596. 23193283 10.1093/nar/gks1219 PMC3531112

[B90] Rabinowitz, H.S., Savage, H.M., Plank, T., Polissar, P.J., Kirkpatrick, J.D., and Rowe, C.D. (2015) Multiple major faults at the Japan Trench: Chemostratigraphy of the plate boundary at IODP Exp. 343: JFAST. Earth Planet Sci Lett 423: 57–66.

[B91] Sakai, S., Takaki, Y., Miyazaki, M., Ogawara, M., Yanagawa, K., Miyazaki, J., et al. (2019) *Methanofervidicoccus abyssi* gen. nov., sp. nov., a hydrogenotrophic methanogen, isolated from a hydrothermal vent chimney in the Mid-Cayman Spreading Center, the Caribbean Sea. Int J Syst Evol Microbiol 69: 1225–1230. 30843780 10.1099/ijsem.0.003297

[B92] Sano, Y., Hara, T., Takahata, N., Kawagucci, S., Honda, M., Nishio, Y., et al. (2014) Helium anomalies suggest a fluid pathway from mantle to trench during the 2011 Tohoku-Oki earthquake. Nat Commun 5: 3084. 24430337 10.1038/ncomms4084

[B93] Sasaki, A., Arikawa, Y., and Folinsbee, R.E. (1979) Kiba reagent method of sulfur extraction applied to isotopic work. Bull Geol Surv Jpn 30: 241–245.

[B94] Schmieder, R., and Edwards, R. (2011) Quality control and preprocessing of metagenomic datasets. Bioinformatics 27: 863–864. 21278185 10.1093/bioinformatics/btr026 PMC3051327

[B95] Shibuya, T., Russell, M., and Takai, K. (2016) Free energy distribution and hydrothermal mineral precipitation in Hadean submarine alkaline vent systems; importance of iron redox reactions under anoxic condition. Geochim Cosmochim Acta 175: 1–19.

[B96] Sim, M.S., Bosak, T., and Ono, S. (2011) Large sulfur isotope fractionation does not require disproportionation. Science 333: 74–77. 21719675 10.1126/science.1205103

[B97] Sugimoto, A., and Wada, E. (1995) Hydrogen isotopic composition of bacterial methane: CO_2_/H_2_ reduction and acetate fermentation. Geochim Cosmochim Acta 59: 1329–1337.

[B98] Summons, R.E., Franzmann, P.D., and Nichols, P.D. (1998) Carbon isotopic fractionation associated with methylotrophic methanogenesis. Org Geochem 28: 465–475.

[B99] Taguchi, K., Yamamoto, T., Nakagawa, M., Gilbert, A., and Ueno, Y. (2020) A fluorination method for measuring the ^13^C-^13^C isotopologue of C_2_ molecules. Rapid Commun Mass Spectrom. 34: e8761. 32067266 10.1002/rcm.8761

[B100] Takai, K., Inoue, A., and Horikoshi, K. (1999) *Thermaerobacter marianensis* gen. nov., sp. nov., an aerobic extremely thermophilic marine bacterium from the 11,000‍ ‍m deep Mariana Trench. Int J Syst Bacteriol 49: 619–628. 10319484 10.1099/00207713-49-2-619

[B101] Takai, K., Inoue, A., and Horikoshi, K. (2002) *Methanothermococcus okinawensis* sp. nov., a thermophilic, methane-producing archaeon isolated from a Western Pacific deep-sea hydrothermal vent system. Int J Syst Evol Microbiol 52: 1089–1095. 12148612 10.1099/00207713-52-4-1089

[B102] Tasumi, E., Yanagawa, K., Miyazaki, J., and Takai, K. (2015) In vitro high-pressure incubation and activity measurement of deep-sea methanogenic archaea. In *Hydrocarbon and Lipid Microbiology Protocols*. *Springer Protocols Handbooks*. McGenity, T., Timmis, K., and Nogales, B. (eds). Berlin, Heidelberg: Springer, pp. 51–64.

[B103] Teske, A. (2020) 10 years of extreme microbiology: an interim reflection and future prospects. Front Microbiol 11: 131. 32117152 10.3389/fmicb.2020.00131 PMC7012785

[B104] Toffin, L., Webster, G., Weightman, A.J., Fry, J.C., and Prieur, D. (2004) Molecular monitoring of culturable bacteria from deep-sea sediment of the Nankai Trough, Leg 190 Ocean Drilling Program. FEMS Microbiol Ecol 48: 357–367. 19712305 10.1016/j.femsec.2004.02.009

[B105] Tostevin, R., Turchyn, A.V., Farquhar, J., Johnston, D.T., Eldridge, D.L., Bishop, J.K.B., et al. (2014) Multiple sulfur isotope constraints on the modern sulfur cycle. Earth Planet Sci Lett 396: 14–21.

[B106] Turner, A.C., Korol, R., Eldridge, D.L., Bill, M., Conrad, M.E., Miller, T.F.III, et al. (2021) Experimental and theoretical determinations of hydrogen isotopic equilibrium in the system CH_4_-H_2_-H_2_O from 3 to 200°C. Geochim Cosmochim Acta 314: 223–269.

[B107] Ueno, Y., Ono, S., Rumble, D., and Maruyama, S. (2008) Quadruple sulfur isotope ana­lysis of ca. 3.5 Ga Dresser Formation: new evidence for microbial sulfate reduction in the Early Archean. Geochim Cosmochim Acta 72: 5675–5691.

[B108] Ujiie, K., Tanaka, H., Saito, T., Tsutsumi, A., Mori, J.J., Kameda, J., et al. (2013) Low coseismic shear stress on the Tohoku-Oki megathrust determined from laboratory experiments. Science 342: 1211–1214. 24311683 10.1126/science.1243485

[B109] Valentine, D.L., Chidthaisong, A., Rice, A., Reeburgh, W.S., and Tyler, S.C. (2004) Carbon and hydrogen isotope fractionation by moderately thermophilic methanogens. Geochim Cosmochim Acta 68: 1571–1590.

[B126] Vinson, D.S., Blair, N.E., Martini, A.M., Larter, S., Orem, W.H., McIntosh, J.C. (2017) Microbial methane from in situ biodegradation of coal and shale: a review and reevaluation of hydrogen and carbon isotope signatures. Chem Geol 453: 128–145.

[B110] Wakita, H., Nakamura, Y., Kita, I., Fujii, N., and Notsu, K. (1980) Hydrogen release: New indicator of fault activity. Science 210: 188–190. 17741286 10.1126/science.210.4466.188

[B111] Walter, S., Laukenmann, S., Stams, A.J.M., Vollmer, M.K., Gleixner, G., and Röckmann, T. (2012) The stable isotopic signature of biologically produced mole­cular hydrogen (H_2_). Biogeosciences 9: 4115–4123.

[B113] Wiersberg, T., and Erzinger, J. (2008) Origin and spatial distribution of gas at seismogenic depths of the San Andreas Fault from drill-mud gas ana­lysis. Appl Geochem 23: 1675–1690.

[B114] Wortmann, U.G., Bernasconi, S.M., and Böttcher, M.E. (2001) Hypersulfidic deep biosphere indicates extreme sulfur isotope fractionation during single-step microbial sulfate reduction. Geology 29: 647–650.

[B115] Wu, S.-Y., and Lai, M.-C. (2011) Methanogenic archaea isolated from Taiwan’s Chelungpu Fault. Appl Environ Microbiol 77: 830–838. 21148697 10.1128/AEM.01539-10 PMC3028716

[B116] Yanagawa, K., Nunoura, T., McAllister, S.M., Hirai, M., Breuker, A., Brandt, L., et al. (2013) The first microbiological contamination assessment by deep-sea drilling and coring by the D/V Chikyu at the Iheya North hydrothermal field in the Mid-Okinawa Trough (IODP Expedition 331). Front Microbiol 4: 327. 24265628 10.3389/fmicb.2013.00327 PMC3820981

[B117] Yanagawa, K., Ijiri, A., Breuker, A., Sakai, S., Miyoshi, Y., Kawagucci, S., et al. (2016) Defining boundaries for the distribution of microbial communities beneath the sediment-buried, hydrothermally active seafloor. ISME J 11: 529–542. 27754478 10.1038/ismej.2016.119 PMC5270560

[B118] Yancey, P., Clark, M., Hand, S., Bowlus, R., and Somero, G. (1982) Living with water stress: evolution of osmolyte systems. Science 217: 1214–1222. 7112124 10.1126/science.7112124

[B119] Yang, T., Dekkers, M.J., and Chen, J. (2018) Thermal alteration of pyrite to pyrrhotite during earthquakes: new evidence of seismic slip in the rock record. J Geophys Res: Solid Earth 123: 1116–1131.

[B120] Yang, Y., Zhang, Y., Cápiro, N. L., and Yan, J. (2020) Genomic characteristics distinguish geographically distributed *Dehalococcoidia*. Front Microbiol 11: 546063. 33013780 10.3389/fmicb.2020.546063 PMC7506110

[B123] Zinke, L.A., Mullis, M.M., Bird, J.T., Marshall, I.P.G., Jørgensen, B.B., Lloyd, K.G., et al. (2017) Thriving or surviving? Evaluating active microbial guilds in Baltic Sea sediment. Environ Microbiol Rep 9: 528–536. 28836742 10.1111/1758-2229.12578

[B124] Zinke, L.A., Glombitza, C., Bird, J.T., Røy, H., Jørgensen, B.B., Lloyd, K.G., et al. (2019) Microbial organic matter degradation potential in Baltic Sea sediments is influenced by depositional conditions and in situ geochemistry. Appl Environ Microbiol 85: e02164–e02118. 30504213 10.1128/AEM.02164-18 PMC6365825

