# Supplementary material for: Deep Subseafloor Biogeochemical Processes and Microbial Populations Potentially Associated with the 2011 Tohoku-oki Earthquake at the Japan Trench Accretionary Wedge (IODP Expedition 343)
Source: Microbes Environ. 2023 Jun 16;38(2):ME22108. doi: 10.1264/jsme2.ME22108 (PMC10308235; doi:10.1264/jsme2.ME22108)
Supplement: Supplementary file 1 — Supplementary Material [file 38_22108_s1.pdf]

Supplementary Table S1. Results of geochemical analyses. Data marked by * is referred from [Proceedings of the Integrated Ocean Drilling Program, Volume 343/343T].

Sample ID	lithU	depth	H_2	CO	CH_4	C_2H_6	pH	Alkalinity	SO_4	Cl	NH_4	d13CO_2	d13CH_4	dDCH_4	dDH_2	dDH_2O	d18OH_2O	d34SO_4sulfate	D33SO_4sulfate	H_4SiO_4
#	#	m	uM	uM	mM	uM	#	meq	mM	mM	uM	permil	permil	permil	permil	permil	permil	permil	permil	uM
1R1	1	177.9	0.4	bdl	8.8	0.184	7.940	49.33	3.63	561	3.21	-2.4	-78.0	-202	NA	1.00	0.14	20.25	0.05	885
1R2	1	179.31	0.7	bdl	3.1	0.097	NA	NA	NA	NA	NA	-1.5	-73.2	-195	NA	NA	NA	NA	NA	NA
1R3	1	180.71	0.1	bdl	15.7	0.3	NA	NA	NA	NA	NA	-1.9	-74.1	-200	NA	NA	NA	NA	NA	NA
1R4	1	181.717	0.2	bdl	5.1	0.134	NA	NA	NA	NA	NA	-1.4	-74.2	-200	NA	NA	NA	NA	NA	NA
1R5	1	183.127	0.1	bdl	4.1	0.131	NA	NA	NA	NA	NA	-3.5	-67.0	-185	NA	NA	NA	NA	NA	NA
1R6	1	184.527	0.1	bdl	1.4	0.078	NA	NA	NA	NA	NA	-3.4	-74.0	-201	NA	NA	NA	NA	NA	NA
2R1	2	649.405	1.9	bdl	0.5	bdl	NA	NA	NA	NA	NA	-11.2	-70.8	-191	NA	NA	NA	NA	NA	NA
2R2	2	650.815	2.4	bdl	1.0	bdl	NA	NA	NA	NA	NA	-8.2	-69.9	-194	NA	NA	NA	NA	NA	NA
3R1	2	654.4	0.2	bdl	0.9	bdl	NA	NA	NA	NA	NA	-6.4	-68.8	-201	NA	NA	NA	NA	NA	NA
3R3	2	657.225	0.2	bdl	1.8	bdl	NA	NA	NA	NA	NA	-4.8	-68.0	-197	NA	NA	NA	NA	NA	NA
3R4	2	658.64	0.5	bdl	1.4	bdl	NA	NA	NA	NA	NA	-8.6	-70.9	-188	NA	NA	NA	NA	NA	NA
4R1	3	689.71	0.5	2.2	1.8	0.183	NA	NA	3.84	562	7.84	-0.4	-66.1	-195	-438	-0.02	0.10	NA	NA	930
4Rcc	3	690.445	32.1	4.4	2.5	0.227	NA	NA	NA	NA	NA	0.2	-65.3	-195	-806	NA	NA	NA	NA	NA
5R1	3	697.4	209.0	1.9	4.0	0.355	NA	NA	NA	NA	NA	-2.4	-65.1	-199	-827	NA	NA	NA	NA	NA
5R2	3	698.69	8.9	3.6	2.8	0.295	NA	NA	3.41	555	7.59	0.1	-65.5	-197	-750	2.44	0.24	NA	NA	885
5R3	3	699.985	2.5	1.8	2.1	0.171	NA	NA	NA	NA	NA	-0.6	-64.4	-198	-511	NA	NA	NA	NA	NA
6R1	3	705.32	1.3	1.2	4.5	0.272	NA	NA	NA	NA	NA	1.9	-65.3	-181	NA	NA	NA	NA	NA	NA
6R2	3	706.15	2.8	2.8	3.4	0.208	NA	NA	8.21	555	7.56	-3.9	-66.6	-195	NA	1.88	-0.06	NA	NA	756
7R2-0	3	714.15	2.4	2.0	8.9	0.358	NA	NA	4.16	558	7.39	-4.7	-64.4	-194	NA	1.32	-0.16	NA	NA	695
7R2-145	3	715.61	4.7	2.0	2.6	0.188	NA	NA	NA	NA	NA	-3.8	-65.5	-183	NA	NA	NA	NA	NA	NA
8R1	3	720.415	2.4	3.8	13.0	0.506	7.910	35.25	6.35	558	6.30	-1.3	-66.2	-189	NA	2.38	0.06	NA	NA	940
8R2	3	721.815	2.0	1.8	7.3	0.409	NA	NA	NA	NA	NA	-8.8	-66.1	-205	NA	NA	NA	19.67	0.0609	NA
8R3	3	722.821	2.0	2.1	6.0	0.311	NA	NA	NA	NA	NA	-7.4	-66.9	-196	NA	NA	NA	NA	NA	NA
9R1	3	725.115	2.2	2.0	15.9	0.578	NA	NA	NA	NA	NA	-8.7	-66.3	-204	NA	NA	NA	NA	NA	NA
10R1	3	771.01	1.6	1.1	4.7	0.222	NA	NA	NA	NA	NA	-4.0	-68.2	-190	NA	NA	NA	NA	NA	NA
10R2	3	771.995	5.0	2.0	1.3	0.116	NA	NA	NA	NA	NA	-7.1	-66.5	-190	-722	NA	NA	NA	NA	NA
11Rcc	3	786	1.1	2.2	20.2	0.628	NA	NA	NA	NA	NA	-5.0	-68.9	-202	NA	NA	NA	NA	NA	NA
12R1	3	786.87	1.5	1.2	9.2	0.4	NA	NA	10.79	570	2.62	-4.5	-69.0	-197	NA	3.82	0.37	NA	NA	660
12R2	3	787.74	2.0	0.9	3.2	0.156	NA	NA	NA	NA	NA	-13.7	-70.6	-204	NA	NA	NA	NA	NA	NA
13R1	3	801.8	2.0	0.7	5.1	0.194	NA	NA	NA	NA	NA	-10.8	-72.1	-196	NA	NA	NA	NA	NA	NA
13R2	3	802.59	2.6	1.7	12.2	0.333	NA	NA	4.97	572	2.83	-14.0	-71.9	-195	NA	2.65	0.18	NA	NA	729
14R1	3	811.01	1.9	1.4	5.9	0.24	NA	NA	3.87	562	1.89	-15.6	-76.1	-193	NA	1.21	-0.13	NA	NA	876
14R2	3	811.64	1.9	0.7	10.6	0.315	NA	NA	NA	NA	NA	-16.8	-75.6	-191	NA	NA	NA	NA	NA	NA
15Rcc-10.5	3	817.825	26.4	bdl	6.8	0.246	NA	NA	4.15	556	1.34	-17.8	-78.5	-184	-795	0.79	0.22	NA	NA	887
15Rcc-20.5	3	817.925	4.2	bdl	8.6	0.235	NA	NA	NA	NA	NA	-21.6	-78.0	-188	NA	NA	NA	NA	NA	NA
16R1-0	3	818.51	1.7	0.6	3.7	0.216	NA	NA	NA	NA	NA	-18.0	-81.2	-180	NA	NA	NA	NA	NA	NA
16R1-119.5	3	819.705	1.2	2.8	6.6	0.282	NA	NA	NA	NA	NA	-20.7	-81.2	-177	NA	NA	NA	NA	NA	NA
16Rcc-0	3	819.715	19.7	2.3	5.5	0.287	NA	NA	NA	NA	NA	-20.7	-80.4	-184	-807	NA	NA	NA	NA	NA
16Rcc-29.5	3	820.01	1.6	3.0	2.6	0.175	NA	NA	NA	NA	NA	-19.2	-81.4	-172	NA	NA	NA	NA	NA	NA
17R1-0	4	821.51	0.5	bdl	3.6	0.123	NA	NA	NA	NA	NA	-22.3	-82.7	-156	NA	NA	NA	NA	NA	NA
17R1-99	4	822.5	1.5	bdl	3.0	0.113	NA	NA	NA	NA	NA	-25.3	-84.0	-163	NA	NA	NA	NA	NA	NA
18R1-0	5	824.01	1.7	bdl	1.8	0.061	NA	NA	NA	NA	NA	-24.1	-83.1	-160	-369	NA	NA	NA	NA	NA
18R1-148.5	5	825.495	1.1	bdl	3.7	0.133	NA	NA	NA	NA	NA	-27.4	-83.6	-161	NA	NA	NA	NA	NA	NA
18Rcc-0	5	825.505	1.3	bdl	2.8	0.105	NA	NA	NA	NA	NA	-26.6	-83.6	-161	NA	NA	NA	NA	NA	NA
18Rcc-17	5	825.675	1.4	bdl	3.2	0.13	NA	NA	NA	NA	NA	-25.5	-83.8	-164	NA	NA	NA	NA	NA	NA
19R1-118	5	827.69	2.3	bdl	1.3	0.135	NA	NA	NA	NA	NA	-28.5	-81.5	-161	NA	NA	NA	NA	NA	NA
19Rcc	5	828.75	0.4	bdl	0.9	bdl	7.640	7.94	16.03	549	0.44	-21.7	-83.1	-167	NA	1.12	0.15	NA	NA	1116
20R1-0	5	831.01	0.8	bdl	0.7	bdl	NA	NA	20.24	544	0.23	-23.0	-82.6	-174	NA	1.72	0.33	NA	NA	1101
20R1	5	832.32	1.1	bdl	0.4	bdl	NA	NA	NA	NA	NA	-25.0	-82.9	-159	NA	NA	NA	23.86	0.0546	NA
20R2	6	833.5	0.8	bdl	0.0	bdl	NA	NA	NA	NA	NA	-18.1	-61.4	-106	NA	NA	NA	NA	NA	NA
SW	SW	835	0	0	0	0	7.8	2.3	28	560	0	NA	NA	NA	NA	0	0	23	0	180
reference			*	*	*	*	*	*	*	*	*	this study	this study	this study	this study	this study	this study	this study	this study	*

Sample ID	Ca	Mg	Sr	Li	K	PO_4	Mn	Br	Ba	B	Fe	V	Zn	Rb	Mo	Cs	Pb	U	Cu
#	mM	mM	uM	uM	mM	uM	uM	uM	uM	uM	uM	nM	nM	nM	nM	nM	nM	nM	nM
1R1	7.20	51.65	107	59	10.65	95.67	2.95	0.93	112.6	576	3.40	145	548	1585	547	3.59	3.75	18.26	1010
1R2	NA	NA	NA	NA	NA	NA	NA	NA	NA	NA	NA	NA	NA	NA	NA	NA	NA	NA	NA
1R3	NA	NA	NA	NA	NA	NA	NA	NA	NA	NA	NA	NA	NA	NA	NA	NA	NA	NA	NA
1R4	NA	NA	NA	NA	NA	NA	NA	NA	NA	NA	NA	NA	NA	NA	NA	NA	NA	NA	NA
1R5	NA	NA	NA	NA	NA	NA	NA	NA	NA	NA	NA	NA	NA	NA	NA	NA	NA	NA	NA
1R6	NA	NA	NA	NA	NA	NA	NA	NA	NA	NA	NA	NA	NA	NA	NA	NA	NA	NA	NA
2R1	NA	NA	NA	NA	NA	NA	NA	NA	NA	NA	NA	NA	NA	NA	NA	NA	NA	NA	NA
2R2	NA	NA	NA	NA	NA	NA	NA	NA	NA	NA	NA	NA	NA	NA	NA	NA	NA	NA	NA
3R1	NA	NA	NA	NA	NA	NA	NA	NA	NA	NA	NA	NA	NA	NA	NA	NA	NA	NA	NA
3R3	NA	NA	NA	NA	NA	NA	NA	NA	NA	NA	NA	NA	NA	NA	NA	NA	NA	NA	NA
3R4	NA	NA	NA	NA	NA	NA	NA	NA	NA	NA	NA	NA	NA	NA	NA	NA	NA	NA	NA
4R1	8.00	42.02	106	98	8.87	27.96	2.44	1.11	104.3	425	1.55	110	1432	1164	929	6.94	26.14	15.88	3630
4Rcc	NA	NA	NA	NA	NA	NA	NA	NA	NA	NA	NA	NA	NA	NA	NA	NA	NA	NA	NA
5R1	NA	NA	NA	NA	NA	NA	NA	NA	NA	NA	NA	NA	NA	NA	NA	NA	NA	NA	NA
5R2	7.73	41.06	109	94	8.49	32.87	2.59	1.11	123.1	482	2.05	109	1101	1091	1018	6.59	37.28	9.65	21165
5R3	NA	NA	NA	NA	NA	NA	NA	NA	NA	NA	NA	NA	NA	NA	NA	NA	NA	NA	NA
6R1	NA	NA	NA	NA	NA	NA	NA	NA	NA	NA	NA	NA	NA	NA	NA	NA	NA	NA	NA
6R2	8.05	41.41	94	86	8.62	22.24	2.22	1.04	32.9	414	0.83	78	2404	1030	1986	5.66	7.90	23.30	3762
7R2-0	8.11	40.91	101	91	9.33	16.63	1.70	1.07	92.2	375	0.93	89	1397	1111	1604	6.32	33.35	13.24	7236
7R2-145	NA	NA	NA	NA	NA	NA	NA	NA	NA	NA	NA	NA	NA	NA	NA	NA	NA	NA	NA
8R1	8.92	43.13	100	88	8.98	22.11	3.06	1.03	68.9	494	1.37	83	1050	1019	735	6.21	12.36	10.51	3186
8R2	NA	NA	NA	NA	NA	NA	NA	NA	NA	NA	NA	NA	NA	NA	NA	NA	NA	NA	NA
8R3	NA	NA	NA	NA	NA	NA	NA	NA	NA	NA	NA	NA	NA	NA	NA	NA	NA	NA	NA
9R1	NA	NA	NA	NA	NA	NA	NA	NA	NA	NA	NA	NA	NA	NA	NA	NA	NA	NA	NA
10R1	NA	NA	NA	NA	NA	NA	NA	NA	NA	NA	NA	NA	NA	NA	NA	NA	NA	NA	NA
10R2	NA	NA	NA	NA	NA	NA	NA	NA	NA	NA	NA	NA	NA	NA	NA	NA	NA	NA	NA
11Rcc	NA	NA	NA	NA	NA	NA	NA	NA	NA	NA	NA	NA	NA	NA	NA	NA	NA	NA	NA
12R1	11.65	44.87	91	74	9.61	NA	5.96	0.93	13.9	325	0.63	87	963	1144	1717	6.59	14.85	14.38	1260
12R2	NA	NA	NA	NA	NA	NA	NA	NA	NA	NA	NA	NA	NA	NA	NA	NA	NA	NA	NA
13R1	NA	NA	NA	NA	NA	NA	NA	NA	NA	NA	NA	NA	NA	NA	NA	NA	NA	NA	NA
13R2	14.53	42.84	104	87	7.95	7.91	11.70	0.96	22.9	297	0.61	56	778	920	1870	5.56	15.94	9.39	3676
14R1	15.77	40.09	102	98	8.19	NA	11.28	0.93	32.9	300	0.58	41	1658	906	623	4.64	8.78	5.89	1814
14R2	NA	NA	NA	NA	NA	NA	NA	NA	NA	NA	NA	NA	NA	NA	NA	NA	NA	NA	NA
15Rcc-10.5	17.94	39.29	109	97	8.56	NA	13.21	0.93	38.6	305	0.71	53	1407	882	80	3.02	15.12	5.94	2720
15Rcc-20.5	NA	NA	NA	NA	NA	NA	NA	NA	NA	NA	NA	NA	NA	NA	NA	NA	NA	NA	NA
16R1-0	NA	NA	NA	NA	NA	NA	NA	NA	NA	NA	NA	NA	NA	NA	NA	NA	NA	NA	NA
16R1-119.5	NA	NA	NA	NA	NA	NA	NA	NA	NA	NA	NA	NA	NA	NA	NA	NA	NA	NA	NA
16Rcc-0	NA	NA	NA	NA	NA	NA	NA	NA	NA	NA	NA	NA	NA	NA	NA	NA	NA	NA	NA
16Rcc-29.5	NA	NA	NA	NA	NA	NA	NA	NA	NA	NA	NA	NA	NA	NA	NA	NA	NA	NA	NA
17R1-0	NA	NA	NA	NA	NA	NA	NA	NA	NA	NA	NA	NA	NA	NA	NA	NA	NA	NA	NA
17R1-99	NA	NA	NA	NA	NA	NA	NA	NA	NA	NA	NA	NA	NA	NA	NA	NA	NA	NA	NA
18R1-0	NA	NA	NA	NA	NA	NA	NA	NA	NA	NA	NA	NA	NA	NA	NA	NA	NA	NA	NA
18R1-148.5	NA	NA	NA	NA	NA	NA	NA	NA	NA	NA	NA	NA	NA	NA	NA	NA	NA	NA	NA
18Rcc-0	NA	NA	NA	NA	NA	NA	NA	NA	NA	NA	NA	NA	NA	NA	NA	NA	NA	NA	NA
18Rcc-17	NA	NA	NA	NA	NA	NA	NA	NA	NA	NA	NA	NA	NA	NA	NA	NA	NA	NA	NA
19R1-118	NA	NA	NA	NA	NA	NA	NA	NA	NA	NA	NA	NA	NA	NA	NA	NA	NA	NA	NA
19Rcc	32.73	36.69	120	101	7.50	NA	164.32	0.86	1.4	426	0.63	33	3443	654	965	2.27	7.85	47	4248
20R1-0	38.94	35.38	121	96	7.06	3.56	113.22	0.87	1.3	410	0.60	36	3267	700	759	1.90	8.74	30	3775
20R1	NA	NA	NA	NA	NA	NA	NA	NA	NA	NA	NA	NA	NA	NA	NA	NA	NA	NA	NA
20R2	NA	NA	NA	NA	NA	NA	NA	NA	NA	NA	NA	NA	NA	NA	NA	NA	NA	NA	NA
SW	11	55	91	26	10	3	0	0.86	150	430	0.1	40	10	1400	107	2.3	0.15	14	6
reference	*	*	*	*	*	*	*	*	*	*	*	*	*	*	*	*	*	*	*

Supplymentary Table S2. Sumarry of the results of microbiogocal experiments; cell concentration in sediment core samples, and quantitative cultivation analysis and activity of radiotracer-incubation analyses using the core samples. NG; Not Growth, ND; Not Detected.

Sample ID		Depth (mbsf)	Cell number (cells/ml-sediment)	Quantitative cultivation analysis		Activity of radiotracer-incubation analyse				
				Population density (cells/g)	Closest phylogenetic relatives [Accession number]	Methanogenesis from H_2/CO_2 (x10-3 g-1 day-1)	Methanogenesis from Acetate (x10-3 g-1 day-1)	Methanogenesis from methylamine (x10-3 g-1 day-1)	Acetogenesis from H_2/CO_2 [mmol g-1 day-1]	Anaerobic methane oxidation [nmol g-1 day-1]
C0019E	1R	177.25	1.8E+05	1.06E+03	*Acetobacterium carbinolicum* [AB546237]	ND	ND	ND	ND	23.8
	4R	689.66	5.3E+05	NG	-	ND	ND	ND	ND	ND
	5R	698.7	5.3E+04	9.17E+02	*Acetobacterium carbinolicum* [AB546237]	ND	ND	ND	7.8	ND
	6R	706.105	6.3E+05	1.06E+03	*Acetobacterium carbinolicum* [AB546237]	ND	ND	ND	ND	18.8
	7R	713.66	below the detection limit	1.06E+03	*Acetobacterium carbinolicum* [AB546237]	ND	ND	ND	ND	ND
	8R	720.76	7.0E+04	1.02E+03	*Acetobacterium carbinolicum*	ND	ND	ND	18.0	ND
	12R	767.7	2.4E+05	ND	-	ND	ND	ND	ND	ND
	13R	802.5	4.2E+05	9.64E+04	*Acetobacterium carbinolicum* [AB546237]<br>*Methanolobus sp.* [LC183835]	ND	ND	0.03	ND	10.8
	14R	810.805	below the detection limit	NG	-	ND	ND	ND	96.9	ND
	15R	816.745	9.5E+04	NG	-	ND	ND	ND	ND	ND
	19R	828.895	4.4E+04	NG	-	ND	ND	ND	ND	ND
	20R	831.415	8.8E+04	NG	-	ND	ND	ND	ND	ND

Supplementary Table S3. Total organic carbon (TOC), total nitogen (TN), and total sulfur (TS) contents in the solid phase and the sulfur isotope composition of Chromium (II)-reducible sulfur (CRS).

Core	Type	Sect	depth	IC	$CaCO_3$	TN	TC	TS	TOC	TOC/TN	TS/TOC	CRS	d34S	34Serror	D33S	33Serror	D36S	36Serror	Remarks
/	/	/	m	wt%	wt%	wt%	wt%	wt%	wt%	/	/	ppm	permil	permil	permil	permil	permil	permil	/
1	R	1	177.4	0.0	0.15	0.05	0.41	0.23	0.40	7.43	0.58	283	-49.982	0.005	0.12	0.004	-0.539	0.051	NA
1	R	6	183.4	0.0	0.15	0.05	0.24	0.08	0.22	4.49	0.38	NA	NA	NA	NA	NA	NA	NA	NA
1	R	6	184.4	0.0	0.15	0.02	0.08	0.01	0.06	3.01	0.19	NA	NA	NA	NA	NA	NA	NA	NA
2	R	1	648.5	0.0	0.27	0.03	0.11	0.02	0.07	2.27	0.23	NA	NA	NA	NA	NA	NA	NA	NA
2	R	1	649.0	0.9	7.49	0.03	0.94	0.00	0.04	1.25	0.13	NA	NA	NA	NA	NA	NA	NA	NA
3	R	1	653.4	0.0	0.38	0.04	0.22	0.06	0.18	4.68	0.31	NA	NA	NA	NA	NA	NA	NA	NA
3	R	1	653.4	0.0	0.30	0.04	0.10	0.08	0.06	1.68	1.33	NA	NA	NA	NA	NA	NA	NA	NA
4	R	1	688.7	1.5	12.29	0.10	2.24	0.30	0.76	7.58	0.39	NA	NA	NA	NA	NA	NA	NA	NA
4	R	1	689.4	3.8	32.07	0.08	4.48	0.15	0.63	7.94	0.23	3607	-37.56	0.004	0.161	0.008	-0.884	0.034	NA
5	R	2	698.2	0.1	0.77	0.10	0.80	0.22	0.71	6.80	0.31	2574	-13.719	0.005	0.03	0.013	-0.115	0.059	NA
6	R	2	705.8	0.1	0.73	0.09	0.57	1.62	0.48	5.63	3.38	11214	-9.584	0.007	0.034	0.004	-0.121	0.044	NA
7	R	1	713.1	0.6	5.39	0.10	1.26	0.22	0.61	6.44	0.36	1229	-23.171	0.004	0.069	0.009	-0.192	0.082	NA
7	R	2	715.4	0.0	0.16	0.10	0.61	0.13	0.59	5.67	0.22	NA	NA	NA	NA	NA	NA	NA	NA
8	R	1	719.5	0.1	1.16	0.10	0.82	0.19	0.68	6.81	0.27	NA	NA	NA	NA	NA	NA	NA	NA
8	R	2	720.9	0.1	0.83	0.11	0.81	0.27	0.71	6.62	0.39	1668	-37.173	0.005	0.156	0.008	-0.564	0.061	NA
9	R	1	724.6	0.0	0.14	0.10	0.60	0.03	0.58	6.13	0.05	NA	NA	NA	NA	NA	NA	NA	NA
10	R	1	770.9	0.1	1.14	0.09	0.77	0.46	0.63	7.24	0.73	NA	NA	NA	NA	NA	NA	NA	NA
10	R	CC	772.2	0.1	1.19	0.08	0.71	0.27	0.56	6.79	0.49	NA	NA	NA	NA	NA	NA	NA	NA
11	R	CC	780.6	0.1	0.55	0.10	0.76	0.21	0.70	7.34	0.30	NA	NA	NA	NA	NA	NA	NA	NA
12	R	2	787.5	0.1	0.56	0.08	0.68	0.19	0.61	8.04	0.30	1312	-32.505	0.007	0.096	0.014	-0.234	0.042	NA
13	R	1	801.3	0.1	1.11	0.09	0.81	0.19	0.68	7.56	0.27	NA	NA	NA	NA	NA	NA	NA	Gray
13	R	1	801.5	0.1	0.70	0.06	0.49	1.03	0.41	7.34	2.54	1399	-15.68	0.004	0.025	0.011	0.129	0.045	Brown
14	R	1	810.5	0.3	2.28	0.09	0.87	3.19	0.60	6.92	5.33	399	-13.345	0.004	0.078	0.011	-0.528	0.056	NA
15	R	1	816.8	0.0	0.05	0.03	0.10	0.11	0.10	3.55	1.15	820	14.779	0.007	0.121	0.006	-1.07	0.039	NA
16	R	1	818.6	0.1	1.06	0.06	0.65	0.27	0.52	8.25	0.51	NA	NA	NA	NA	NA	NA	NA	NA
17	R	1	822.6	0.0	0.17	0.03	0.05	0.04	0.03	1.05	1.25	NA	NA	NA	NA	NA	NA	NA	NA
18	R	1	824.6	0.0	0.06	0.04	0.12	0.06	0.11	2.81	0.54	NA	NA	NA	NA	NA	NA	NA	NA
19	R	2	828.4	0.1	0.44	0.03	0.16	0.03	0.10	3.90	0.31	NA	NA	NA	NA	NA	NA	NA	NA
20	R	1	832.3	0.1	0.54	0.04	0.18	0.03	0.11	3.10	0.26	NA	NA	NA	NA	NA	NA	NA	NA
20	R	2	833.0	0.0	0.27	0.01	0.09	0.04	0.06	5.54	0.80	NA	NA	NA	NA	NA	NA	NA	NA
20	R	2	833.1	0.0	0.13	0.01	0.08	0.05	0.06	5.63	0.76	NA	NA	NA	NA	NA	NA	NA	NA
20	R	2	833.5	0.0	0.12	0.01	0.05	0.03	0.04	5.09	0.81	NA	NA	NA	NA	NA	NA	NA	NA

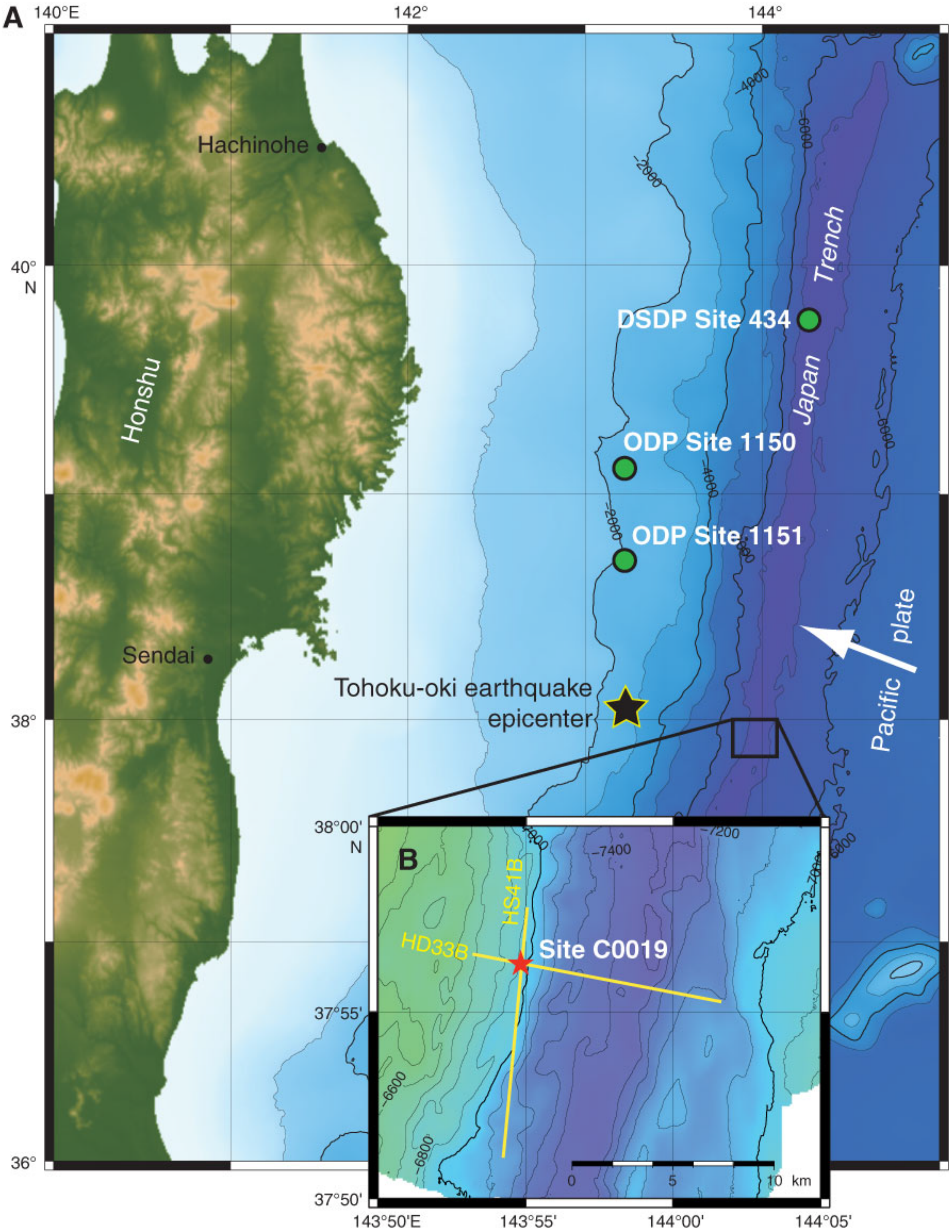

Fig S1. A;Maps of the sampling Site C0019 (rad star) and previously drilled DSDP and ODP sites (green circles). The black star indicated the Tohoku-oki epicenter, and white arrow showed Pacific plate convergence vector. B; Close up Site C0019.

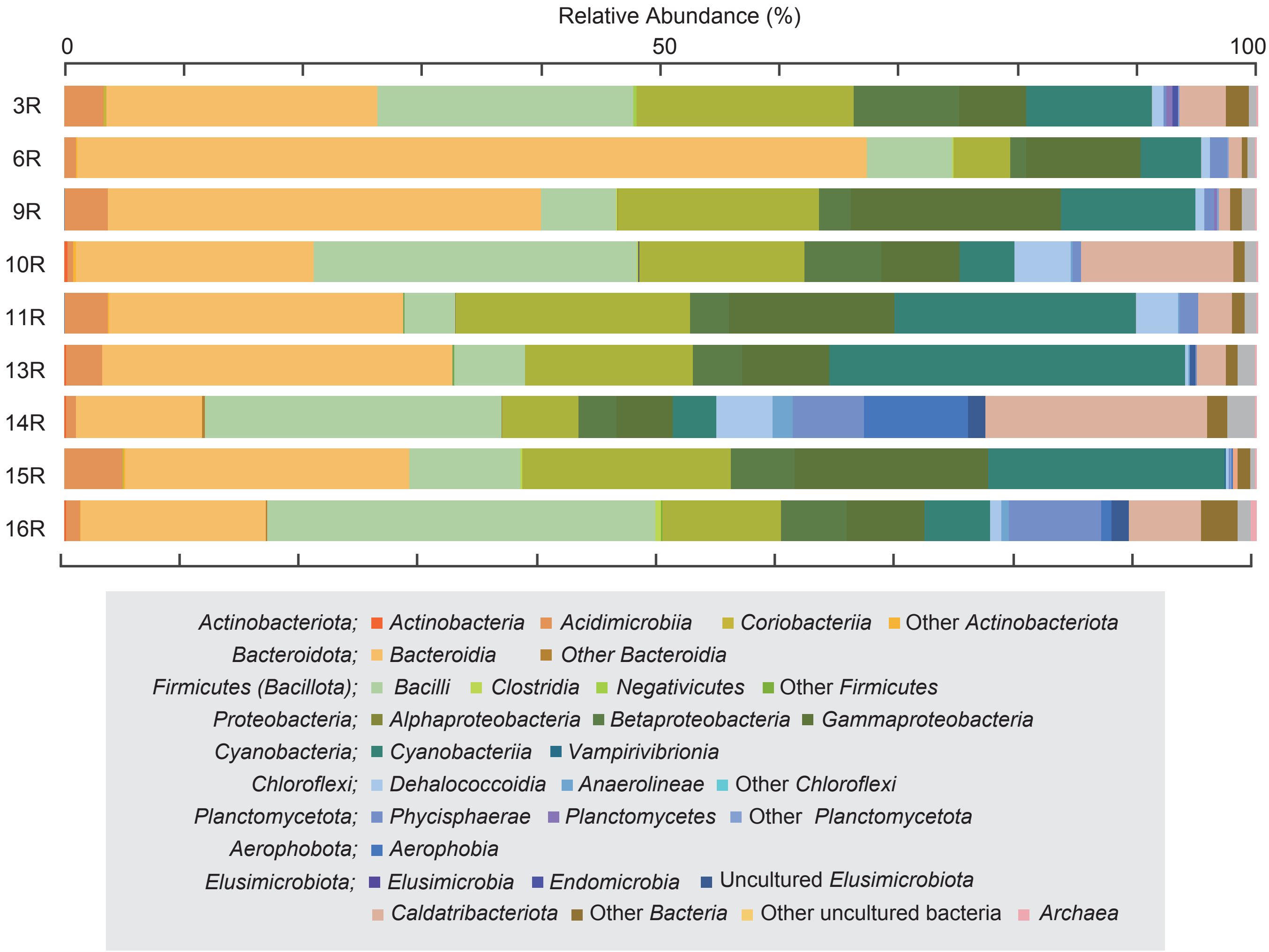

Fig. S2. Summary of microbiological analyses.
Taxonomic composition of 16S rRNA gene amplicon sequences in sediments at site C0019E.
Class-level taxonomic composition based on SSU rRNA gene amplicon sequencing using the universal primers.
